# Demethylation at enhancer upregulates MCM2 and NUP37 expression predicting poor survival in hepatocellular carcinoma patients

**DOI:** 10.1186/s12967-022-03249-2

**Published:** 2022-01-29

**Authors:** Zengwei Tang, Yuan Yang, Wen Chen, Enliang Li, Tingbo Liang

**Affiliations:** 1 Department of Hepatobiliary and Pancreatic Surgery, The First Affiliated Hospital, School of Medicine, Zhejiang University, 79 Qingchun Road, Hangzhou, 310003 Zhejiang China; 2 Zhejiang Provincial Key Laboratory of Pancreatic Disease, The First Affiliated Hospital, School of Medicine, Zhejiang University, Hangzhou, 310003 Zhejiang China; 3 Zhejiang Provincial Innovation Center for the Study of Pancreatic Diseases, Zhejiang Province, Hangzhou, 310003 Zhejiang China; 4 Department of Hematology, Peking Union Medical College Hospital, Chinese Academy of Medical Science and Peking Union Medical College, Beijing, 100730 China; 5 Cancer Center, Zhejiang University, Hangzhou, 310058 Zhejiang China; 6 Research Center for Healthcare Data Science, Zhejiang Lab, Hangzhou, 310003 Zhejiang China

**Keywords:** Hepatocellular carcinoma, Biomarkers, Survival, Epigenetic regulation, Methylation

## Abstract

**Background:**

Identification of novel biomarker is important for development of molecular-targeted therapy agents for patients with hepatocellular carcinoma (HCC). This study aims to identify potential prognostic biomarkers and investigate epigenetic mechanism of HCC development.

**Methods:**

Public bulk-RNA seq datasets and proteomic dataset were screened for identification of potential prognostic biomarkers for HCC patients. Public methylomic datasets were analyzed for deciphering the epigenetic mechanism regulating HCC-associated gene expression. Immunoblotting, immunohistochemistry, real-time PCR, and pyrosequencing were used to validate the findings from bioinformatic analyses.

**Results:**

Minichromosome maintenance complex component 2 (MCM2) and nucleoporin 37 (NUP37) were overexpressed in human HCC tissues and hepatoma cell lines. MCM2 significantly positively correlated with NUP37 expression. Higher expression of MCM2 or NUP37 was significantly associated with advanced tumor stage and worse overall survival in 3 large independent HCC cohorts (n = 820). MCM2 and NUP37 overexpression are independent prognostic risk factors for HCC patients. Demethylation at an enhancer of MCM2 gene was a common event in patients with HCC, which significantly negatively correlated with MCM2 and NUP37 mRNA expression.

**Conclusions:**

Demethylation at enhancer regulates MCM2 and NUP37 expression in HCC. MCM2 and NUP37 are promising prognostic biomarkers and potential targets for epigenetic therapy in HCC patients.

**Supplementary Information:**

The online version contains supplementary material available at 10.1186/s12967-022-03249-2.

## Background

Hepatocellular carcinoma (HCC) is the most common primary hepatic malignancy, accounting for ~ 90% of all primary liver cancers, which is the third most common cause of cancer-related deaths worldwide [1]. Most HCC patients tend to have an underlying liver disease, such as hepatitis B or C, alcohol abuse disorder or non-alcoholic steatohepatitis. Therefore, efficacy of the current therapeutic approaches is limited [2]. In addition, the prognosis of HCC remains dismal due to the lack of effective non-surgical treatment options, especially for patients at the advanced stage. Programmed cell death protein 1 (PD-1) immune checkpoint inhibitors have been approved for treatment of HCC. However, this was accompanied by a high rate of adverse events and low treatment response in patients with advanced disease [3]. Sorafenib, the first FDA-approved, molecular-targeted drug for advanced-stage HCC, unfortunately has limited survival benefits. However, tyrosine kinase inhibitor ramucirumab has showed markedly survival benefits in HCC patients with elevated serum α-fetoprotein (AFP) after previous treatment with sorafenib [4].

TP53, CTNNB1 and ALB were identified as the most frequently mutated loci in HCC [5–7]. Furthermore, a recent study, investigating the mutation landscapes in HCC, identified PYCR2 and ADH1 as significant prognostic biomarkers that are involved in the metabolic reprogramming of HCC cells [8]. However, these mutated gene signatures are yet to be translated so far into clinically relevant biomarkers. This is mainly due to the lack of druggable targets, considerable genetic and epigenetic heterogeneities in HCC. Aberrant DNA methylation has been associated with oncogenesis in human cancers including HCC [9–11]. Recent evidence showed that promoter hypomethylation increases ITPR3 expression while promoter hypermethylation reduce OGDHL expression in HCC [10, 11], which indicates that epigenetic regulation plays an important role in HCC development and there are promising epigenetic therapy targets for HCC patients.

Identification of novel causative molecular biomarkers would contribute to understand the molecular events in HCC development, which can potentially optimize the clinical therapy of HCC patients. Determination of epigenetic mechanism regulating potential oncogene expression in HCC would aid in identifying potential drug targets for development of novel epigenetic therapy agents. Therefore, we performed an integrated analysis of the transcriptomic, proteomic and methylomic datasets of HCC. We further validated the expression profiles of minichromosome maintenance complex component 2 (MCM2) and nucleoporin 37 (NUP37) in both clinical HCC samples and human HCC cells. Furthermore, we determined the prognostic role of MCM2 and NUP37 by analysis of three large independent HCC cohorts (n = 820). In addition, we investigated and validated the epigenetic mechanisms upregulating MCM2 and NUP37 expression in HCC.

## Methods

### Public data sources and bioinformatic analysis

Public bulk-RNA seq datasets TCGA-LIHC (https://portal.gdc.cancer.gov/projects/TCGA-LIHC), GSE57957 (https://www.ncbi.nlm.nih.gov/geo/query/acc.cgi?acc=GSE57957), and E-MTAB-4171 (https://www.ebi.ac.uk/arrayexpress/experiments/E-MTAB-4171/), including 421 HCC samples and 104 paired nontumor liver samples, were downloaded for identification of differentially expressed genes (DEGs) at mRNA levels. Public proteomic dataset OEP000321 (https://www.biosino.org/node/project/detail/OEP000321) including 159 pairs HCC and adjacent nontumor liver samples were downloaded from the National Omics Data Encyclopedia (NODE) for identification of differentially expressed proteins (DEPs). Moreover, public methylomic datasets E-MTAB-4169 (https://www.ebi.ac.uk/arrayexpress/experiments/E-MTAB-4169/), TCGA-LIHC (https://portal.gdc.cancer.gov/projects/TCGA-LIHC), and GSE56588 (https://www.ncbi.nlm.nih.gov/geo/query/acc.cgi?acc=GSE56588), including 638 HCC samples, 93 paired nontumor liver samples, 10 normal liver samples and 9 cirrhotic liver samples, were analyzed for determining the methylation patterns of potentially identified biomarkers for HCC patients.

The R package “TCGAbiolinks” [12] was used to download and process the raw data of TCGA-LIHC datasets. Differentially expressed genes (DEGs) as well as differentially expressed proteins (DEPs) between the HCC and nontumor tissues were identified using the ‘limma’ R package [13] based on *P* value < 0.05 and |logFC| $\ge$ 2 and 1, respectively. In addition, differential methylation regions (DMRs) were identified among CpG or non-CpG islands throughout the genome scale. The “minfi” and “limma” R packages [13, 14] were used for identification of DMRs with *P* value < 0.05 and |logFC| $\ge$ 0.2.

### Patients and tissue specimens

We prospectively collected 10 HCC and adjacent tissue (peritumor) samples from primary HCC patients without receiving neoadjuvant treatment after curative surgical therapy from May to July 2020 at the First Affiliated Hospital of Zhejiang University, Hangzhou, China. The collected each fresh specimen was snap-frozen in liquid nitrogen, and then used for protein and RNA extraction, or stored at −80 °C for subsequent experiments. Moreover, two sets of tissue microarrays (TMAs), generated in-house, including paraffin-embedded tissues from 300 primary HCC patients who had undergone surgical resection from 2010 to 2018 in our hospital, were used for immunohistochemistry (IHC). All patients were followed-up routinely every 3 to 6 months after surgery. The clinicopathological information on these 300 cases of HCC patients was summarized in Additional file 1: Table S1. Written informed consents were obtained from each patient in this study. The experiment protocol for use of clinical samples was approved by Institutional Ethics Committee in First Affiliated Hospital of Zhejiang University.

### Reagents and antibodies

Anti-MCM2 antibody (HPA031496), anti-NUP37 antibody (ab220675), anti-GAPDH antibody (ab181602), Goat Anti-Rabbit IgG H&L (ab205718) and DAB Substrate Kit (ab64238) were purchased from Sigma and Abcam, respectively. Human MCM2, NUP37 and β-actin primer sets were purchased from Gene Chem (Shanghai, CN). Decitabine (S1200) and CPI-455 (S8287) were purchased from Selleck (Shanghai, CN). Dimethyl sulfoxide (DMSO) was purchased from Sigma.

### Cell culture

Human HCC cell lines Huh7, Hep3B, HepG2, Bel-7402, HCC-LM3, normal liver cell line HL7702, and mouse hepatoma cell line Hep1-6 were purchased from The Cell Bank of Chinese Academy of Sciences (Shanghai, China) with authentication between 2018 and 2020. HL7702, Huh7, and Bel-7402 were cultured in high glucose DMEM (Gibco), other cells were cultured in MEM (Gibco) supplemented with 10% FBS, 100 μg/ml streptomycin (Gibco), and 100 U/mL penicillin (Gibco) at 37 °C in 5% CO2 atmosphere. All cell lines had been tested regularly for mycoplasma contamination. In addition, 3 × 10^5^ Huh7 cells per well were planted in 6-well cell culture plate and incubated for 48 h in high glucose DMEM supplemented with 10% FBS and the indicated concentration of decitabine or CPI-455. Decitabine was dissolved with phosphate buffered saline (PBS) and CPI-455 was dissolved with DMSO.

### Cell viability assay

Cell viability was assessed using a CCK8 assay (Beyotime, Shanghai, CN). Briefly, Hep3B cells (1 × 10^4^ cells per well) were seeded into 96-well plates and incubated in antibiotic-free growth medium supplemented with decitabine, CPI-455, PBS or DMSO. After incubated for 24, and 48 h, fresh complete medium with CCK-8 (1:10) was added to each well, and the cells were incubated at 37 °C in 5% CO_2_ atmosphere for 1 h. Finally, the absorbance at 450 nm was detected using a microplate reader (800 TS, BioTek, USA).

### Immunoblotting

Soluble proteins from fresh HCC and paired peritumor tissue were obtained after following procedure: (1) manually grinding tissue into powder within 2 min using a clean mortar after immersed it into liquid nitrogen; (2) then collecting the tissue powder (5–10 mg) into a pre-cold Eppendorf tube containing 300 μl RIPA lysis buffer (Beyotime, Shanghai, CN) supplemented with 1 × protease inhibitor buffer (#5871, Cell Signaling Technology, CN) on ice; (3) constantly agitating the mixture on an orbital shaker at 4 °C atmosphere for 2 h; (4) After centrifugation at 16,000*g* for 20 min at 4 °C, the supernatants were collected. For normal liver cells and HCC cells, washing two times with ice-cold PBS, and lysis with 100 μl RIPA buffer supplemented with protease inhibitor cocktail for 30 min on ice. After centrifugation at 12,000*g* for 15 min at 4 °C, the supernatants were collected as the whole cellular protein extracts. Both tissue and cellular soluble protein extracts were quantified using the bicinchoninic acid protein assay kit (Beyotime, Shanghai, CN).

A total of 30 μg protein was resolved on SDS-PAGE (10% polyacrylamide), transferred to polyvinylidene difluoride (PVDF) membranes. Membranes were blocked with 5% skim milk for 1 h at room temperature, then incubated with anti-MCM2 antibody (0.1 μg/ml), anti-NUP37 (1:2000) or anti-GAPDH antibody (1:5000) overnight at 4 °C. After 3 times washes of membranes in 1× tris-buffered saline with tween 20 (TBST), the membranes were incubated with anti-rabbit immunoglobulin G-horseradish peroxidase-linked secondary antibody (1:3000) for 2–3 h at 4 °C. The protein bands were visualized by a Chemiluminescence Dectection Kit for HRP according to the manufacture’s instruction and detected using ChemiScope 6100 Touch system (CliNX, CN).

### RNA isolation and real-time PCR

Total RNA from human cells or tissues was extracted using Trizol reagent (Life Technologies) according to the instruction of the user manual. The concentration of RNA was determined by spectrophotometry at 260 nm using Nanodrop One (Thermo SCIENTIFIC). The purity of sample was determined by the 260/280 nm ratio. The detected ratio, ranging from 1.8 to 2.1, indicating that the extracted RNA was fine and suitable for subsequent experiments. cDNAs were generated from 1 μg RNA of each samples by using PrimeScript™ RT reagent Kit with gDNA Eraser (Takara, Japan). Real-time PCR (RT-PCR) analysis was performed using TB Green ™ Premix Ex Taq™ (Tli RNaseH Plus, Japan). A final volume of 20 μl mixture was amplified by RT-PCR using ABI QuantStudio-5 Real-Time PCR System (Applied Biosystems by Thermo Fisher Scientific). Reaction conditions were as follows: 95 °C for 30 s, followed by 40 cycles of denaturation at 95 °C for 5 s and annealing at 60 °C for 30 s. The relative mRNA expression was determined using the ΔΔCT method. The primer sequences used in real-time PCR are as follows: MCM2, forward 5’-CTACCAGCGTATCCGAATCCA-3’ and reverse 5’-CCTACAGCAACCTTGTTGTCCT-3’; NUP37, forward 5’-CTGCGTTTCGTGACCTTGTC-3’ and reverse 5’-TACACGTGCCAATGACCACA-3’; β-actin, forward 5’-CGACAGGATGCAGAACGAGA-3’ and reverse 5’-GACCCTGGATGTGACAGCTC-3’.

### Immunohistochemistry (IHC)

Immunohistochemical staining was performed as described previously [15]. After anti-NUP37 antibody (1:200) and anti-MCM2 antibody (1:200) were incubated with tissue sections and TMAs overnight at 4 °C, the biotinylated secondary antibody (1:300) was applied before visualization using DAB substrate. The IHC reaction result was calculated by multiplying a percentage score and staining intensity score. The percentage score was used to describe the estimated percentage of positively stained neoplastic cells (0: 0–5%; 1: 6–25%; 2: 26–50%; 3: 51–75%; 4: > 75%). The intensity score described the estimated staining degree (0: negative; 1: weak; 2: moderate; 3: strong). Both the percentage score and staining score were evaluated independently by two pathologists who were blinded to the research design. Any discrepancy wound be resolved by a consensus among these two pathologists after a re-assessment. The NUP37 score and MCM2 score were used to stratify patients into low and high groups according to the optimal cut-off value.

### Pyrosequencing

For validation of the methylation pattern of MCM2 enhancer region and NUP37 promoter region, genomic DNA from frozen HCC tissues and paired nontumor tissues samples (n = 6) were extracted using Blood and Tissue Kit (#69504, Qiagen). The concentration of genomic DNA from each sample was determined using NANO Quant infinite M200PRO (TECAN) and a total of 500 ng genomic DNA (A260/A280 ratios ranging from 1.8 to 2.0) from each sample was used for bisulfite DNA conversion using the EpiTect Bisulfite Kit (#59104, Qiagen) following the manufacturer’s handbook for user. After cleanup of bisulfite converted genomic DNA, we amplified the target sequence using ABI 9700 PCR System and performed quantitative pyrosequencing using the PyroMark Q96 ID (QIAGEN). The genomic DNA methylation percentage was calculated with using Pyro Q—CpG software (Biotage). The primer sequences used in PCR amplification and pyrosequencing, as well as the chromosomal location of each target sequence in this study were summarized in Additional file 1: Table S2. Pyrosequencing was performed using the platforms from Geneland Biotech Co., Ltd (Shanghai, CN).

### Experimental animal model

To establish subcutaneous implantation model that was used to assess the impact of decitabine on HCC progression in vivo, 3 × 10^5^ Hep1-6 cells (per mouse) in the logarithmic phase, resuspended in 100 μl PBS, were injected into the flanks of 5-week-old male C57BL/6 J mice (Model Animal Research Centre of Nanjing University, Nanjing, CN). Tumor growth was monitored daily using a digital caliper. Upon tumor reached 150 to 300 mm^3^, mice were randomized into control (PBS) or decitabine (3 mg/kg, 3 times weekly) groups with each group involving 5 mice. The mice were administrated with decitabine or PBS by intraperitoneal injection. After the treatment, each mouse body weight was monitored daily using an electronic balance. Mice were sacrificed according to the Institutional Animal Care & Use Committee (IACUC) protocol and subcutaneous tumor lesions were completely excised with using the dissection instruments in a mouse surgical kit. We calculated the tumor volume as follows: Volume = length × (width)^2^ × π/6.

All animal experiments were performed in accordance with the National Institutes of Health’s *Guide for the Care and Use of Laboratory Animals.* The animals were housed in a specific-pathogen-free room with a 12-h light/dark schedule in the facility of the First Affiliated Hospital, Zhejiang University School of Medicine. All animals were fed an autoclaved water and standard food ad libitum, and routine husbandry procedures and daily care were provided by animal care staffs of the facility. All experimental animals were routinely monitored for their general health status, and the experimental protocol was reviewed and approved by IACUC.

### Statistical analysis

All statistical analyses were performed with using R version 4.0.3 for Windows 64-bit. Data are presented as arithmetic mean ± Standard Deviation (SD). ANOVA, chi-square test, fisher's exact test, Student’s t test, or Wilcoxon test was used to determine the statistical significance according to variable type and distribution. The “surv_cutpoint ()” function, encoded in the “survminer” R package (https://cran.r-project.org/web/packages/survminer/index.html) was used to determine the optimal cut-off point for continuous variables that correspond to the survival of HCC patients. Overall survival (OS) analysis was performed using the Kaplan–Meier method, and survival differences were determined by log-rank test encoded in “Survival” R package (https://cran.r-project.org/web/packages/survival/index.html). Multivariate survival analysis was performed using the Cox proportional hazards regression model. Spearman or Pearson correlation analyses were performed to determine the relationship between NUP37 and MCM2 expression, MCM2 or NUP37 mRNA expression and DNA methylation levels, respectively. Differences or correlations with *p* values < 0.05 were considered statistically significant.

## Results

### Identification of DEGs and DMRs in the HCC tissues

**Fig. 1:**
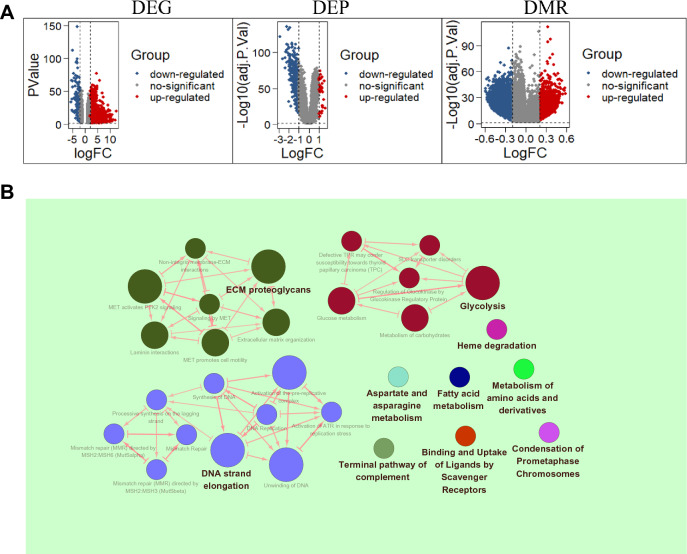
Differential and enrichment analysis results. **A** Volcano plots showing the differentially expressed genes (DEGs), differentially expressed proteins (DEPs), and differentially methylated regions (DMRs) in HCC patients. **B** The top 10 biological processes enriched by DEGs matched with DEPs

Briefly, a total of 1915 differentially expressed genes (DEGs) were identified between the HCC and nontumor tissues by mining the transcriptomic dataset (Fig. 1A). Out of those, 1730 genes were upregulated and 185 were downregulated in HCC. In the proteomic dataset, 38 significantly upregulated and 330 downregulated proteins were identified in HCC compared to nontumor (Fig. 1A). Additionally, 5779 upregulated and 46,906 downregulated methylated cytosine-phosphate-guanine (CpG) or non-CpG islands were present throughout the HCC genomes (Fig. 1A). DNA strand elongation, ECM proteoglycans, and glycolysis were the top three biological processes enriched by the identified DEGs matched with DEPs (Fig. 1B). After assessing the results of reactome pathways enrichment analysis with ClueGO version 2.5.6. (Fig. 1B) and a comprehensive literature review, we subsequently focused on MCM2 and NUP37 that significantly enriched in DNA strand elongation and glycolysis process, respectively.

### MCM2 and NUP37 are upregulated in human HCC

**Fig. 2:**
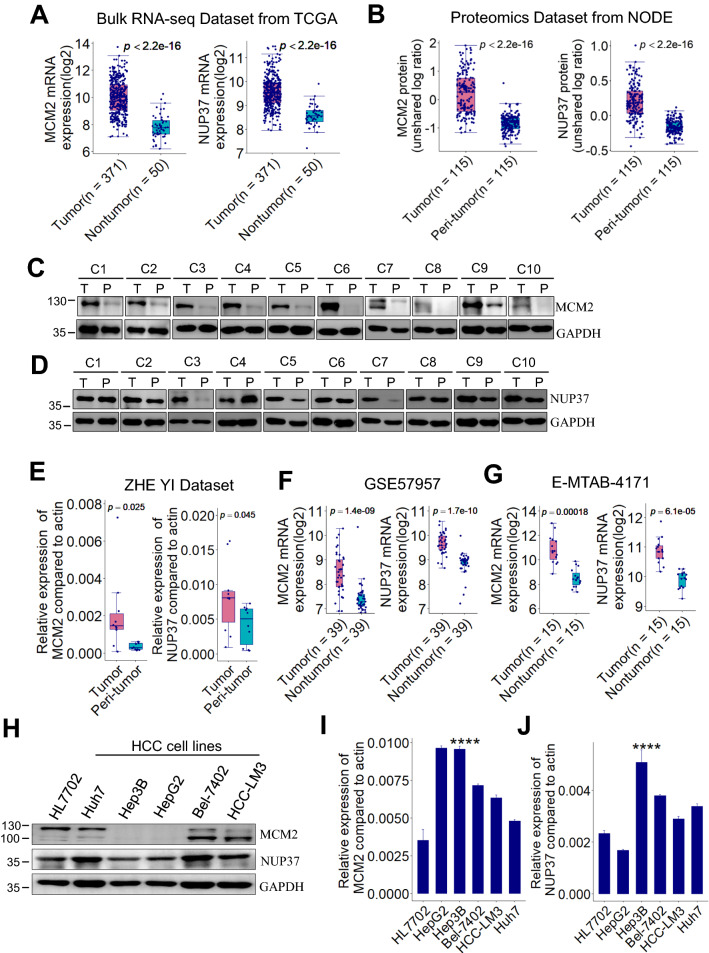
MCM2 and NUP37 are upregulated in human HCC. **A** Box plots showing MCM2 and NUP37 mRNA expression levels between HCC and nontumor samples from TCGA dataset (TCGA-LIHC, n = 421). **B** Box plots showing MCM2 and NUP37 protein expression levels between HCC and peri-tumor samples from NODE dataset (OPE00321, n = 330). *p* value is based on Wilcoxon test. **C**, **D** Representative immunoblot of 10 pairs of HCC sample lysates showing that MCM2 and NUP37 were overexpressed in HCC. **E** MCM2 and NUP37 mRNA expression levels were determined by RT-PCR in 10 pairs of HCC samples. * *p* < 0.05, ** *p* < 0.01 by paired Student’s t-test. **F**, **G** Box plots showing MCM2 and NUP37 mRNA expression levels between HCC and the paired nontumor tissues samples from GSE57957 and E-MTAB-4171 dataset respectively. **H**–**J** MCM2 and NUP37 expression were detected by immunoblot and RT-PCR in human HCC cells and normal liver cells. HCC, hepatocellular carcinoma; TCGA, **** *p* < 0.0001, based on ANOVA. TCGA, The Cancer Genome Atlas; LIHC, liver hepatocellular carcinoma; NODE, National Omics Data Encyclopedia; RT-PCR, real-time PCR

Results from bulk RNA-seq dataset of TCGA showed that MCM2 and NUP37 mRNA levels in HCC tissues are significantly upregulated in comparison with that in nontumor tissues (Fig. 2A). Similarly, the MCM2 and NUP37 protein levels in HCC tissues are significantly higher than that in peri-tumor tissues from NODE proteomics dataset (Fig. 2B). Consistently with the expression profiles in omics datasets of HCC, validation by western blot (Fig. 2C, D) and RT-PCR (Fig. 2E) in 10 pairs of human HCC tissue samples showed that MCM2 and NUP37 were significantly upregulated in HCC relative to the paired peri-tumor samples. H&E staining was performed to assess the histopathology type of each HCC tissue and paired peri-tumor tissue (Additional file 1: Fig. S1). Similar with the findings from our internal validation dataset, results from two external validation datasets also showed that MCM2 and NUP37 mRNA are significantly overexpressed in HCC relative to the paired nontumor tissue samples (Fig. 2F, G).

We also assessed the expression profiles of MCM2 and NUP37 in 5 different hepatoma cell lines and one normal liver cell line (HL7702). Results from western blot showed that MCM2 protein was highly expressed in HL7702, Huh7, Bel-7402 and HCC-LM3 cells compared with that in HepG2 or Hep3B cells (Fig. 2H). Meanwhile, NUP37 protein was determined in all the indicated cells (Fig. 2H). Results from RT-PCR showed that MCM2 mRNA expression levels were higher in the indicated hepatoma cell lines than that in normal liver HL7702 cells (Fig. 2I). In comparison with the expression levels in HL7702 cells, NUP37 mRNA levels were upregulated in the indicated hepatoma cell lines except for HepG2 cells (Fig. 2J).

### MCM2 significantly positively correlated with NUP37 expression in human HCC

**Fig. 3:**
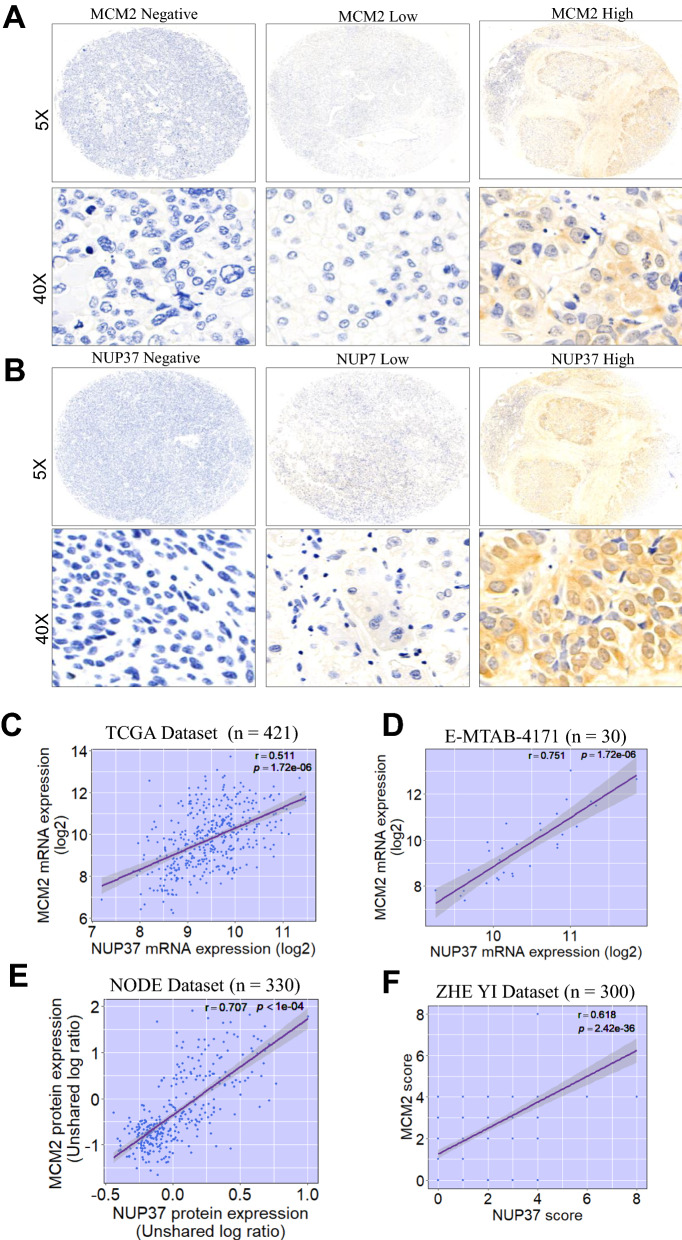
Immunochemistry (IHC) staining of MCM2 or NUP37 in HCC tissues, and the correlation of MCM2 with NUP37 expression in human HCC. **A**–**B** Representative IHC staining of MCM2 or NUP37 in primary HCC sample. **C**–**F** Scatter plots showing MCM2 expression significantly positively correlated with NUP37 expression in 4 independent HCC datasets. Pearson or Spearman correlation analysis was used accordingly based on the data distribution. HCC, hepatocellular carcinoma; TCGA, The Cancer Genome Atlas; NODE, National Omics Data Encyclopedia

IHC staining of HCC tissue samples showed that MCM2 and NUP37 were mainly expressed in nucleoplasm of carcinoma cells (Fig. 3A, B). MCM2 mRNA expression levels were significantly correlated with NUP37 mRNA expression levels in patients with HCC from TCGA dataset (Fig. 3C) and ArrayExpress dataset (Fig. 3D). Similarly, MCM2 protein expression was significantly correlated with NUP37 protein expression in HCC patients from NODE dataset (Fig. 3E). This significantly positively relationship between MCM2 and NUP37 expression profile was further validated by IHC staining in our internal HCC cohort (Fig. 3F).

### MCM2 or NUP37 overexpression predicated worse clinical outcomes for HCC patients

**Fig. 4:**
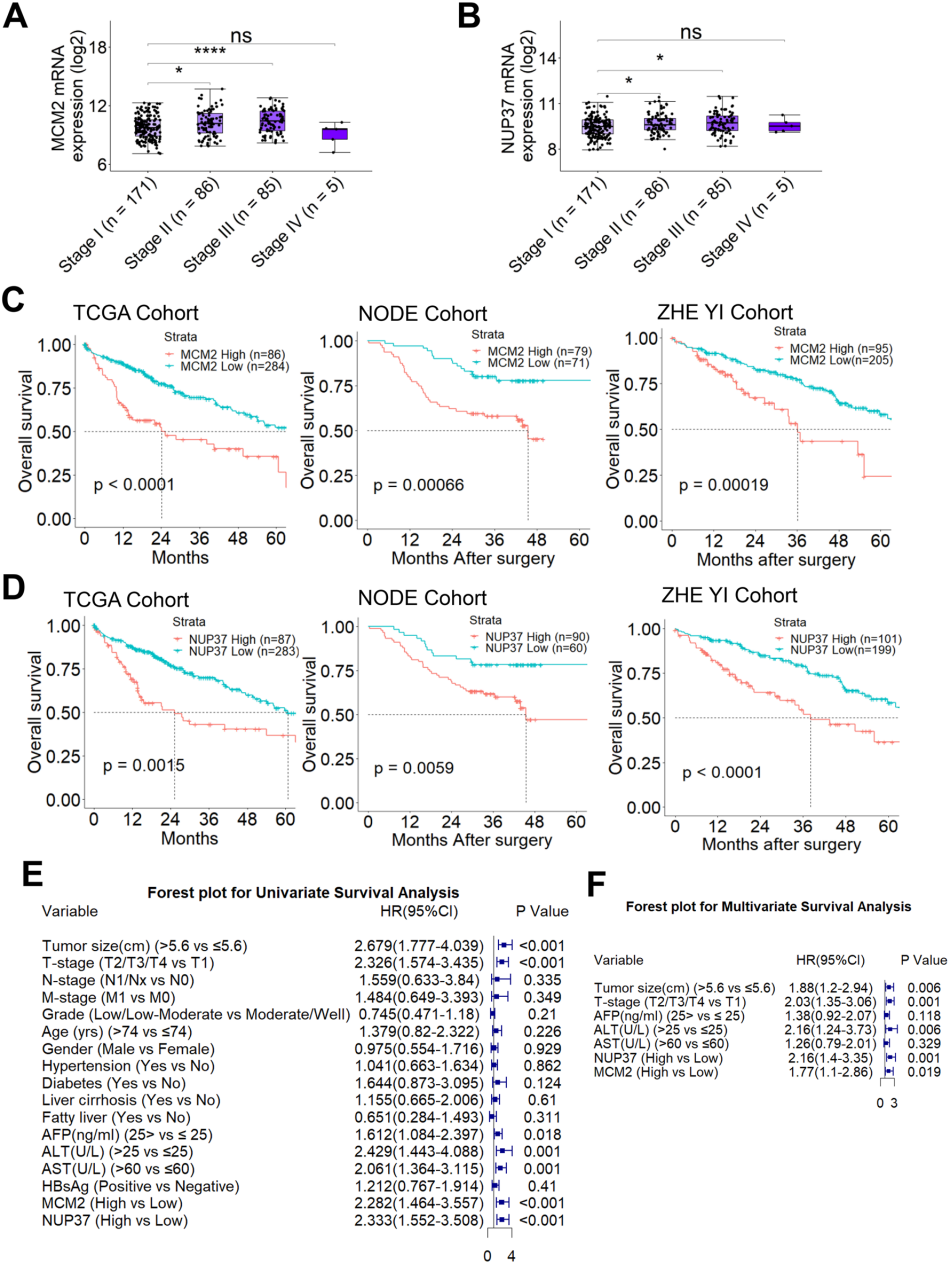
MCM2 and NUP37 overexpression correlated with worse survival in HCC patients. **A**–**B** Box plots showing mRNA of MCM2 or NUP37 significantly correlates with the clinical stage of HCC. * *p* < 0.05, *** *p* < 0.001, based on Wilcoxon test. **C**–**D** Kaplan–Meier survival analysis was performed to assess the impact of MCM2 and NUP37 on survival of HCC patients from 3 independent cohorts. *p* is based on log-rank test. TCGA cohort (n = 370); NODE cohort (n = 150), and ZHE YI Cohort (n = 300). **E**–**F** Forest plots showing Cox univariate and multivariate survival analyses results in ZHE YI Cohort

To determine the clinical relevance of MCM2 or NUP37 overexpression in human HCC, we firstly investigated the correlation between tumor stage (TNM stage) and MCM2 or NUP37 overexpression using TCGA samples. The mRNA expression profiles of MCM2 or NUP37 stratified by the tumor stage showed that MCM2 (Stage II vs. Stage I, *p* < 0.05; Stage III vs. Stage I, *p* < 0.001) or NUP37 (Stage II vs. Stage I, *p* < 0.05; Stage III vs. Stage I, *p* < 0.05) expression was significantly increased in advanced tumor stage (Fig. 4A, B).

Univariate survival analysis was performed to assess the impact of MCM2 or NUP37 expression on the survival of HCC patients from 3 large independent HCC cohorts (n = 820). Based on the optimal cut-off value of 7.623 (log_2_ expression) for MCM2 mRNA, HCC patients from TCGA cohort were stratified into two groups. Kaplan–Meier survival curve showed that HCC patients with high MCM2 mRNA levels had worse OS (median OS, 24.1 (13.8 -NA) months vs. 71.0 (51.0–104.0) months; log-rank, 4.124; *p* < 0.0001, log-rank test; Fig. 4C). Similarly, OS curve showed that patients with high NUP37 mRNA levels (log_2_ expression = 6.914) had worse OS (median OS, 25.2 (14.2 -NA) months *vs.* 60.6 (50.3–94.6) months; *p* = 0.0015, log-rank test; Fig. 4D). HCC patients from NODE proteomic cohort were divided into two groups according to an optimal threshold value of -0.047 (unshared log ratio), and Kaplan–Meier survival curve showed that HCC patients with high MCM2 protein levels had worse OS (median OS, 33.9 (29.8–37.9) months *vs.* 70.7 (64.4–76.9) months;* p* = 0.00066, log-rank test; Fig. 4C). Similarly, Kaplan–Meier survival curve showed that patients with high NUP37 protein levels (unshared log ratio $\ge$ 0.124) had poor OS (median OS, 51.8 (43.4–60.3) months *vs.* 59.4 (53.7–65.0) months; *p* = 0.0059, log-rank test; Fig. 4D).

Notably, these findings were further validated by analysis of our in-house HCC cohort (n = 300). Based on the score of MCM2 or NUP37 determined by IHC, these 300 cases of HCC patients were classified into MCM2 or NUP37 high and low groups. OS analysis showed that HCC patients with MCM2 or NUP37 high expression had worse OS (median OS, 37.3 (29.2–45.3) months vs. 59.5 (53.4–65.6) months; *p* = 0.00019, log-rank test; Fig. 4C; median OS, 35.4 (27.9–42.8) months vs. 60.6 (54.2–67.0) months; *p* < 0.0001, log-rank test; Fig. 4D), respectively. Moreover, Cox univariate survival analysis showed that HCC patients with tumor size > 5.6 cm, advance tumor stage (T4/T3/T2), AFP > 25 ng/ml, ALT > 25U/L, or AST > 60U/L significantly associated with worse OS (Fig. 4E).

Correlation analyses showed that there was no significant relationship between MCM2 or NUP37 expression and the comorbidities of HCC patients (Table 1). Meanwhile, correlation analyses of MCM2 or NUP37 expression with clinicopathological features revealed a significant association between NUP37 overexpression and tumor metastasis (Table 2). In addition, multivariate survival analysis showed that tumor size > 5.6 cm, advanced tumor stage, ALT > 25U/L, MCM2 or NUP37 overexpression were independent risk factors for worse OS of HCC patients (Fig. 4F).

**Table 1:** Relationship between comorbidities and NUP37 or MCM2 expression in HCC patients (n = 300)

Variable	NUP37			MCM2		
	Low	High	*p*-value	Low	High	*p*-value
Hypertension						
No	153	71	0.338	159	65	0.121
Yes	46	30		46	30	
Diabetes						
No	183	89	0.384	187	85	0.787
Yes	16	12		18	10	
Liver cirrhosis						
No	176	93	0.233	185	84	0.781
Yes	23	8		20	11	
Fatty liver						
No	182	86	0.140	184	84	0.883
Yes	17	15		21	11	
Portal vein hypertension						
No	185	89	0.233	188	86	0.906
Yes	14	12		17	9	
Hepatitis B virus						
No	59	20	0.806	56	23	0.895
Yes	160	61		153	68	

HCC, hepatocellular carcinoma

**Table 2:** Relationship between clinicopathological features and NUP37 or MCM2 expression in HCC patients (n = 300)

Variable	NUP37				MCM2		
	Low	High		*p*-value	Low	High	*p*-value
Tumor size							
≤ 5.6 cm	144	69		0.552	151	62	0.176
> 5.6 cm	55	32			54	33	
T-stage							
T1/T2/T3	177	92		0.707	184	85	1.000
T4	22	9			21	10	
N-stage							
N0	195	96		0.172	198	93	0.799
N1/Nx	4	5			7	2	
M-stage							
M0	195	94		0.039	197	92	1.000
M1	4	7			8	3	
Age (year)							
≤ 70	167	85		1.000	172	80	0.946
> 70	32	16			33	15	
Gender							
Female	28	11		0.554	29	10	0.495
Male	171	90			176	85	
AFP (ng/ml)							
≤ 500	156	77		0.782	156	77	0.418
> 500	43	24			49	18	
ALT (U/L)							
≤ 40	131	64		0.768	135	60	0.745
> 40	68	37			70	35	
AST (U/L)							
≤ 60	154	78		0.957	159	73	1.000
> 60	45	23			46	22	

HCC, hepatocellular carcinoma; AFP, α-fetoprotein; ALT, alanine aminotransferase; AST, aspartate aminotransferase

### Demethylation at enhancer upregulated MCM2 expression in HCC

**Fig. 5:**
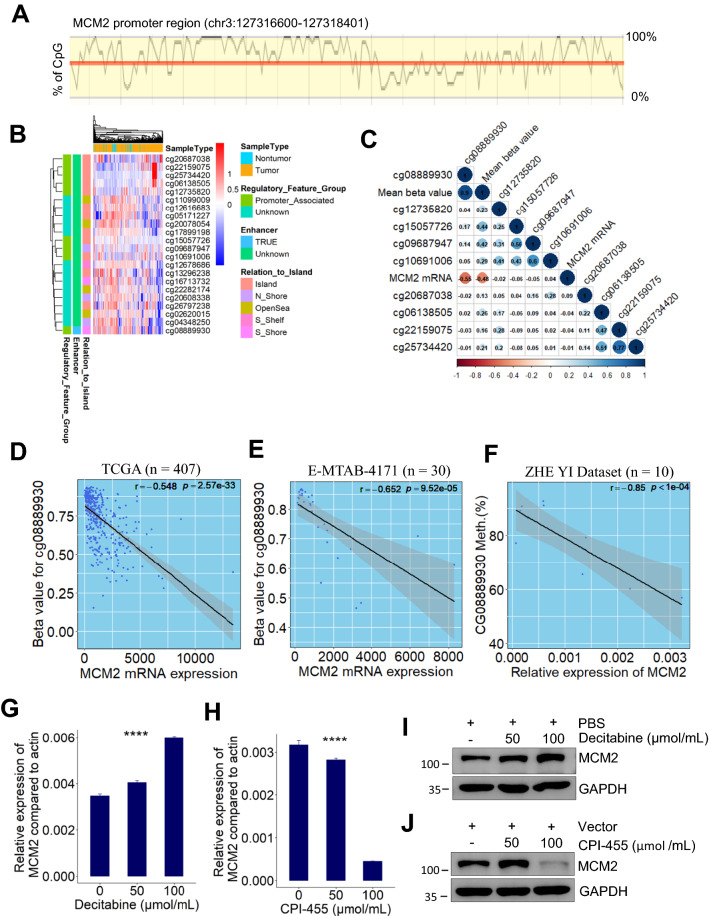
Demethylation at enhancer significantly negatively correlated with MCM2 mRNA expression. **A** Diagram showing the CpG islands in the promoter region of human MCM2 gene. **B** Heatmap showing overall methylation patterns in the DNA sequence of human MCM2 gene. **C** Correlation heatmap visualizing the relationship between MCM2 mRNA expression and the methylation level of CpG site at MCM2 promoter region. **D**–**F** Scatter plot showing that MCM2 mRNA expression significantly negatively correlated with the methylation level of MCM2 enhancer region (cg08889930) in HCC patients from 3 independent datasets. **G** MCM2 mRNA expression in Huh7 cells significantly increased after decitabine treatment **** *p* < 0.0001, based on ANOVA. **H** MCM2 mRNA expression in Huh7 cells significantly reduced after CPI-455 treatment. **** *p* < 0.0001, based on ANOVA. **I**–**J** Representative immunoblot of HCC cells (Huh7) lysates showing that decitabine increased MCM2 protein expression and CPI-455 attenuated MCM2 protein expression. TCGA, The Cancer Genome Atlas; ANOVA, analysis of variance; HCC, hepatocellular carcinoma

To investigate the epigenetics mechanisms upregulating MCM2 expression in HCC patients, we analyzed the methylation profiles of MCM2 gene sequences. Bioinformatic analysis using online ensemble project showed that MCM2 promoter region has some CpG islands (Fig. 5A), which can potentially silence gene expression by DNA methyltransferases-mediated methylation. Nine promoter-associated CpG sites (Fig. 5B) were identified with using “IlluminaHumanMethylation450kanno.ilmn12.hg19” R package (http://bioconductor.org/packages/IlluminaHumanMethylation450kanno.ilmn12.hg19/). By integrative analysis of TCGA bulk RNA-seq and methylomic datasets of HCC patients, we found that DNA methylation levels of MCM2 enhancer (cg08889930) was significantly negatively correlated with MCM2 mRNA expression (Pearson correlation test; r = − 0.548, *p* = 2.57e−33, Fig. 5C, D). This significant relationship was also observed in both one external dataset (Pearson correlation test; r = − 0.652, *p* = 9.52e−05; Fig. 5E) and our internal validation dataset (Pearson correlation test; r = − 0.85, *p* < 0.001; Fig. 5F).

To determine the direct role of DNA methylation in MCM2 expression, decitabine was used to inhibit DNA methyltransferase in vitro. Results from RT-PCR showed that MCM2 mRNA expression levels in Huh7 cells significantly increased after decitabine treatment (Fig. 5G). To investigate whether MCM2 overexpression can be silenced by inhibition of histone demethylation, CPI-455, an inhibitor of histone lysine demethylase 5 (KDM5), was used to suppress the demethylation of histone H3 on lysine 4 (H3K4) in Huh7 cells. Interestingly, results from RT-PCR showed that MCM2 expression significantly downregulated after CPI-455 therapy (Fig. 5H). These findings were further validated by western blot (Fig. 5I, J), which indicating that inhibition of DNA methyltransferase can induce MCM2 expression via downregulating methylation levels of MCM2 gene. Furthermore, inhibition of histone demethylase can significantly attenuate MCM2 expression by inhibition of KDM5 activity.

**Fig. 6:**
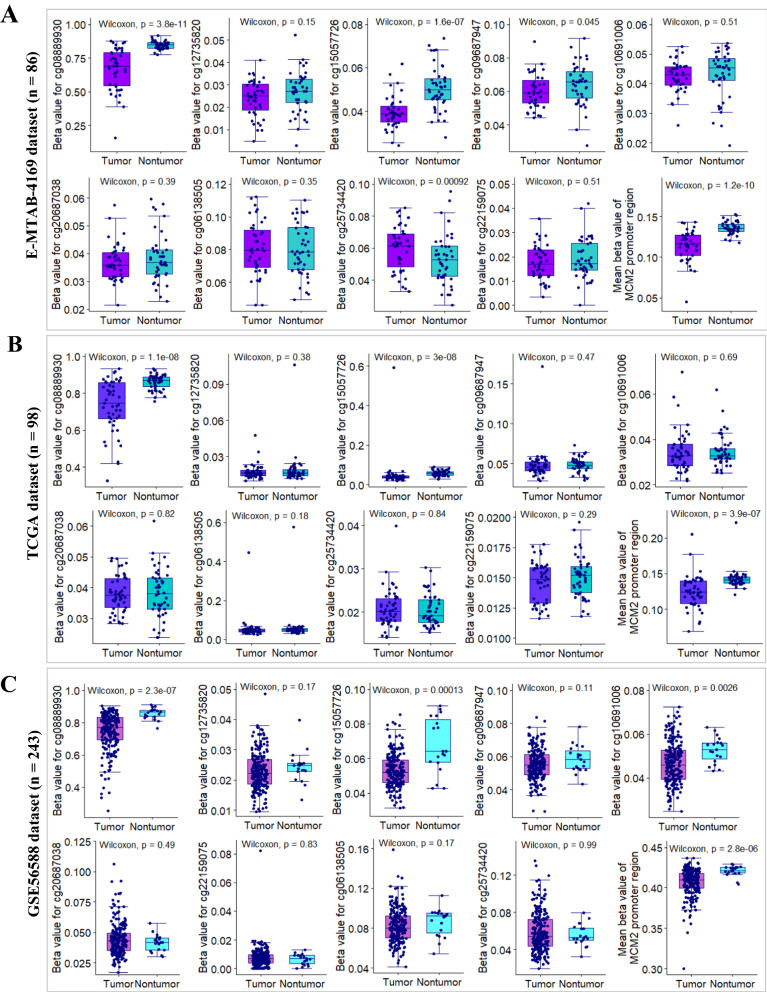
Deciphering DNA methylation pattern of MCM2 promoter region between HCC and nontumor. **A**–**B** Box plots showing methylation patterns CpG islands at the promoter region of MCM2 gene between HCC and paired nontumor samples from E-MTAB-4169 and TCGA datasets, respectively. **C** Box plots showing methylation patterns of CpG islands at MCM2 promoter region of HCC samples (n = 224) and nontumor samples (n = 19) from GSE56588 dataset

To precisely decipher DNA methylation profile of MCM2 promoter region, we analyzed the methylation patterns of CpG islands or CpG island shores in HCC as compared with adjacent nontumor tissue samples from 3 independent HCC datasets with same sequencing platform (Illumina Infinium HumanMethylation450 BeadChip). Results from E-MTAB-4169 showed that there are 4 significantly differential methylation regions between HCC samples and paired adjacent samples (Fig. 6A). Of these 4 regions, the methylation levels of cg08889930 and cg15057726 sites were significantly lower in HCC samples (n = 43) than in nontumor samples (n = 43). Interestingly, in comparison with other CpG sites in promoter region, cg08889930, an enhancer of MCM2 gene, located at CpG island shores, with a mean betavalue > 0.6, was obviously hypermethylated in both tumor and paired nontumor samples (Figs. 5B,  6A). The obvious demethylation patterns of cg08889930 occurred in HCC relative to paired nontumor samples was further confirmed by using another two external validation datasets (Fig. 6B, C) and our internal validation dataset (Additional file 1: Fig. S2A). To assess the impact of aberrant methylation of cg08889930 on the prognosis of HCC patients, HCC patients from TCGA cohort were stratified into two groups according to the optimal threshold value (β-value = 0.556). Kaplan–Meier survival curve showed that HCC patients with lower methylation degree of cg08889930 had significant poor OS (median OS, 15.1 (10.0–28.7) months *vs.* 81.9 (56.5–104.2) months; *p* < 0.0001, log-rank test; Additional file 1: Fig. S2).

Together, these findings indicated that demethylation at enhancer of MCM2 was a common event in HCC patients, which was responsible for MCM2 overexpression in HCC by regulating MCM2 transcription at genome-wide.

### Demethylation at enhancer upregulated NUP37 expression in HCC

**Fig. 7:**
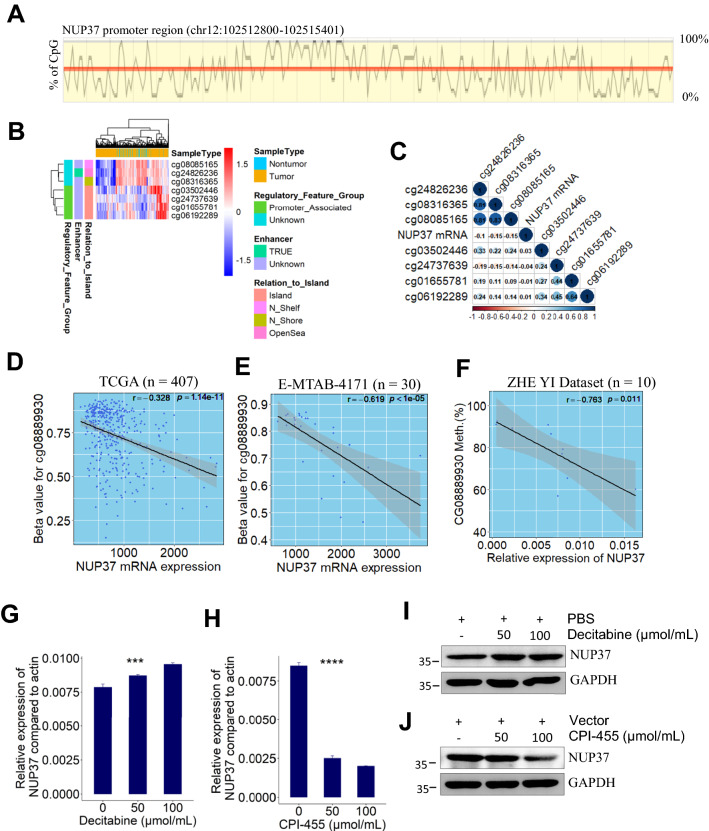
Demethylation at enhancer region (cg08889930) significantly negatively correlated with NUP37 mRNA expression. **A** Diagram showing the CpG islands in the promoter region of human NUP37 gene. **B** Heatmap showing overall methylation patterns in the DNA sequence of human NUP37 gene. **C** Correlation heatmap visualizing the relationship between NUP37 mRNA expression and the methylation level of CpG island at DNA sequence of NUP37 gene. **D**–**F** Scatter plot showing that NUP37 mRNA expression significantly negatively correlated with the methylation level of cg08889930 in HCC patients from 3 independent datasets. **G** NUP37 mRNA expression in Huh7 cells significantly increased after decitabine treatment *** *p* < 0.001, based on ANOVA. **H** NUP37 mRNA expression in Huh7 cells significantly reduced after treatment with CPI-455. **** *p* < 0.0001, based on ANOVA. **I**–**J** Representative immunoblot of HCC cells (Huh7) lysates showing that decitabine increased NUP37 protein expression and CPI-455 decreased NUP37 protein expression

Similarly, CpG islands in NUP37 promoter region (Fig. 7A) are capable of silence NUP37 expression via DNA methyltransferases-mediated methylation. Meanwhile, a total of 6 CpG sites, including 4 promoter-associated CpG sites and 3 promoter-unassociated CpG sites, were identified using “IlluminaHumanMethylation450kanno.ilmn12.hg19” R package (Fig. 7B). To determine the correlation of DNA methylation levels with NUP37 mRNA expression, we performed an integrative analysis of TCGA bulk RNA-seq and methylomic datasets of HCC patients. However, no moderate/strong correlations were determined between NUP37 mRNA expression and the methylation degree of CpG islands/non-CpG islands (Fig. 7C). We hypothesized that the demethylation patterns of cg08889930 indirectly regulates NUP37 expression since NUP37 shares a similar expression pattern with MCM2 at both mRNA and protein levels. Then, by analysis of the relationship between NUP37 mRNA expression and the methylation level of cg0889930, we found that DNA methylation degree of cg08889930 was significantly negatively correlated with NUP37 mRNA expression (Pearson correlation test; r = − 0.328, *p* = 1.14e−11, Fig. 7D). Notably, this finding was finally verified by using one external validation dataset (Pearson correlation test; r = − 0.619, *p* < 1e−05; Fig. 7E) and our internal validation dataset (Pearson correlation test; r = − 0.763, *p* = 0.011; Fig. 7F).

To investigate the impact of DNA methylation on NUP37 expression, decitabine was used to treat HCC cells by inhibition of DNA methyltransferase in vitro. Results from RT-PCR showed that NUP37 mRNA expression levels in Huh7 cells significantly increased after treatment with decitabine (Fig. 7G). To further investigate whether NUP37 expression can be downregulated by inhibition of histone demethylation, CPI-455 was used to treat Huh7 cells. Results from RT-PCR showed that NUP37 expression significantly downregulated after CPI-455 therapy (Fig. 7H). These findings were further validated by western blot (Fig. 7I, J). Taken together, our results showed that inhibition of DNA methyltransferase increased NUP37 expression. Whereas inhibition of histone demethylase decreased NUP37 expression by inhibiting KDM5 activity.

**Fig. 8:**
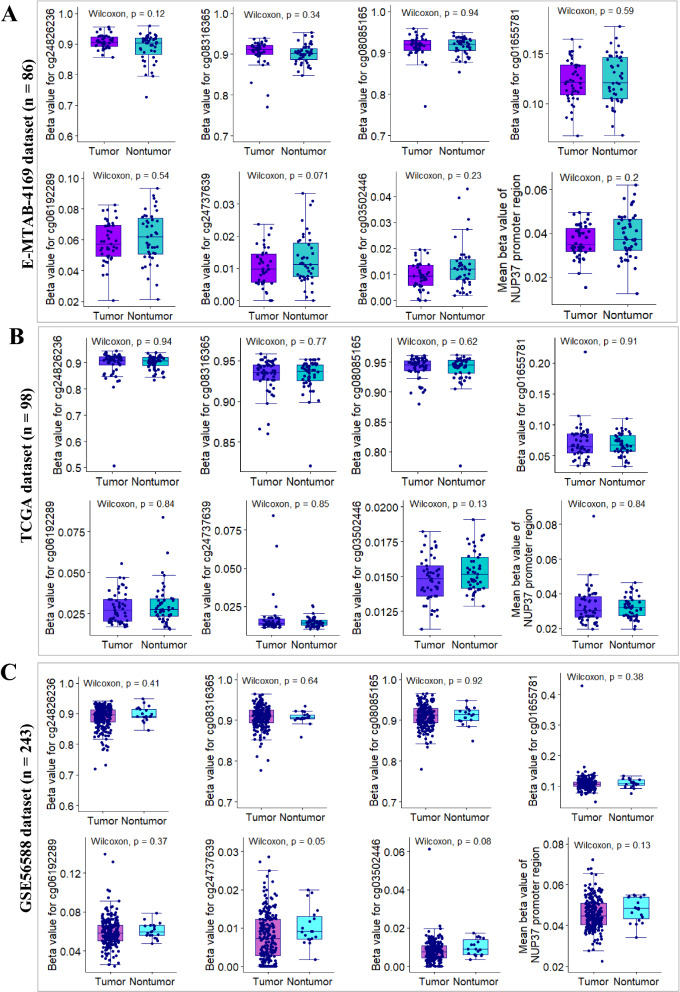
Deciphering DNA methylation pattern of human NUP37 gene between HCC and nontumor. **A**–**B** Box plots showing methylation patterns CpG islands at DNA sequence of NUP37 gene between HCC and paired nontumor samples from E-MTAB-4169 and TCGA datasets, respectively. **C** Box plots showing methylation patterns of CpG islands at DNA sequence of NUP37 gene between HCC samples (n = 224) and nontumor samples (n = 19) from GSE56588 dataset

To get a more precise understanding of DNA methylation patterns of NUP37 gene at genome-wide scale, we also analyzed the methylation profiles of CpG sites in HCC as compared with adjacent nontumor tissue samples from 3 independent HCC datasets with same sequencing platform. Results from E-MTAB-4169 showed that the methylation levels of the indicated CpG sites in HCC samples (n = 43) is similar with that in paired adjacent nontumor samples (Fig. 8A). Consistently with the findings from E-MTAB-4169 dataset, results from another two external validation datasets showed that there are no significantly differential methylation sites between clinical HCC samples and adjacent nontumor samples (Fig. 8B, C). Furthermore, results from our internal validation dataset showed that the methylation pattern of cg03502446 island within NUP37 promoter region is similar between HCC and adjacent nontumor tissues (Additional file 1: Fig. S3B). Interestingly, the methylated percentage of CpG sites of cg24737639 and NUP37 promoter region are significantly lower in HCC tissues than in paired nontumor tissues (Additional file 1: Fig. S3C, D). Importantly, results from both external and internal validation datasets showed that the mean beta-value of NUP37 promoter region was less than 0.2, which indicates that a hypomethylation pattern of NUP37 promoter occurred in both HCC and nontumor tissue samples (Fig. 8). Together, these findings suggested that the possibility of other methylation sites at genome-wide beyond NUP37 location-scale are indirectly responsible for NUP37 mRNA overexpression in HCC patients.

### Epigenetic drugs treatment

**Fig. 9:**
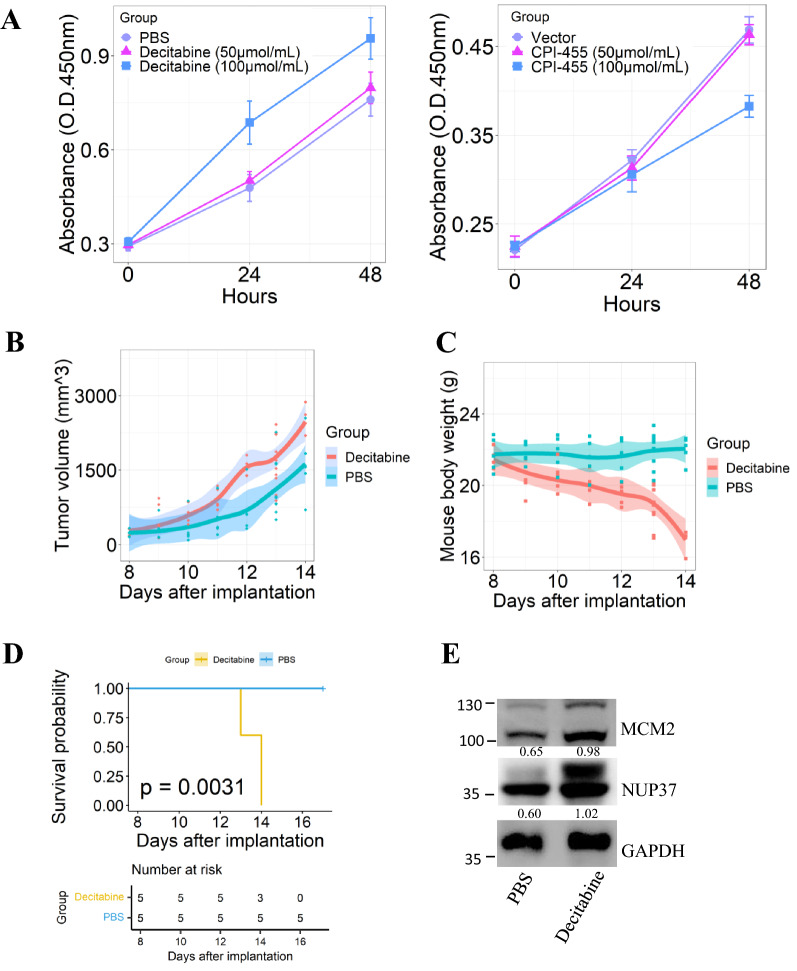
The therapy effect of epigenetic drugs on HCC cells progression. **A** The impact of decitabine and CPI-455 on the proliferation of HCC cells was assessed by CCK8 assay respectively. **B** Decitabine increased the growth of already developed subcutaneous tumors. 3 mg/kg decitabine was administrated by intraperitoneal injection at day8, day10 and day12 into C57BL/6 J mice after implantation. **C** Decitabine decreased mouse body weight. **D** The overall survival of mice was significantly poor in decitabine treatment group relative to the controls. *p* is based on log-rank test. **E** MCM2 and NUP37 expression in tumors from decitabine or PBS group was analyzed by immunoblotting. Values represent the relative MCM2 or NUP37 expression levels compared to GAPDH

Cell viability assays showed that DNA methyltransferase (DNMT) inhibitor decitabine promoted HCC cells proliferation, whereas KDM5 inhibitor CPI-455 inhibited HCC cell proliferation (Fig. 9A). To investigate the effect of decitabine on HCC progression in vivo, we treated tumor-bearing C57BL/6 J mice with decitabine by intraperitoneal injection. We observed that subcutaneous tumor volumes were significantly increased in decitabine treatment group compared to control group (Fig. 9B). Mouse body weight and mouse survival rate were markedly decreased in decitabine group relative to controls (Fig. 9C, D). In addition, MCM2 and NUP37 expression were increased in decitabine-treated group relative to the controls (Fig. 9E). These findings indicated that decitabine can promote HCC progression via upregulating methylation-induced oncogene MCM2 and NUP37 expression. Due to CPI-455 is water-insoluble, we therefore did not assess its treatment effect in vivo.

## Discussion

To identify novel prognostic markers and potential epigenetic therapy targets for primary HCC patients, we performed an integrative analysis of primary HCC-associated multi-omics datasets. Transcriptomic analysis revealed a total of 1915 DEGs between HCC and nontumor. Meanwhile, proteomic analysis only identified 368 DEPs occurred in HCC development when compared with nontumor samples. These findings indicated that there are a lot of molecular events occurred in HCC development and there are many potential drug targets for precision treatment of HCC. Moreover, the huge gap between the DEPs and DEGs for HCC implied that there are complex post-transcriptional modification mechanisms regulating DEGs mRNA translation. Methylation frequently occurred in DNA sequences with a high density of cytosine-guanine dinucleotides, which plays a significant role in maintaining chromosomal stability [16]. In our work, methylomic analysis revealed that demethylation frequently occurred in HCC development, which possibly upregulates HCC-associated gene expression at transcriptional level.

MCM2, one member of the highly conserved minichromosome maintenance complex (MCM) protein family, initiates eukaryotic genome replication and promotes symmetric inheritance of modified histones during DNA replication [17]. MCM2 has been identified as a new biomarker for predicting tumor cell proliferation and prognosis of various cancers [18, 19]. Although several recent studies with small sample size reported that MCM2 upregulated in HCC and promotes HCC cell proliferation [20, 21], -lack of evidence elucidating the epigenetic mechanism regulating MCM2 expression in human HCC. In our present work, firstly, we comprehensively revealed and validated the expression profile of MCM2 in both clinical HCC samples and human hepatoma cell lines. Then we confirmed that MCM2 overexpression in primary HCC tissues was significantly associated with worse OS in 3 large independent HCC cohorts (Fig. 4C). Furthermore, multivariate survival analysis further showed that MCM2 upregulation was an independent risk factor for OS of HCC patients (Fig. 4F). Interestingly, MCM2 protein was lowly detected in HepG2 and Hep3B cell lines (Fig. 1H) whereas the relative expression level of MCM2 transcript was higher in these two cell lines relative to the remaining cells (Fig. 1I), which may be caused by some potential post-transcriptional modifications occurred more frequently that inhibit specific translation processes of MCM2 mRNA in HepG2 and Hep3B cells.

Aberrant DNA methylation has been correlated with oncogenesis of multiple cancers [9]. DNA hypomethylation of promoter regions played a key role in regulating ITPR3 gene expression in human HCC [11]. In the present work, we firstly elaborated the demethylation profiles of MCM2 promoter region in HCC compared with nontumor samples. Moreover, we found that DNA methylation degree of MCM2 enhancer (cg08889930) was responsible for regulating MCM2 expression in HCC. Our work also determined that demethylation at MCM2 enhancer is a common event in human HCC development. DNA methyltransferase (DNMT) inhibitors azacitidine and decitabine, antimetabolites that can inhibit DNMT activity and induce hypomethylation when incorporated into DNA, have been approved to treat myelodysplastic syndrome or leukemia [22, 23]. In our study, we showed that decitabine can upregulate MCM2 expression by inducing hypomethylation in MCM2 genome-scale. Inversely, we also found that MCM2 expression was attenuated in HCC cells after treatment with KDM5 inhibitor CPI-455. Together, these finding implied that MCM2 is a potential epigenetic therapy target for HCC patients.

NUP37, also known as p37 or MCPH24, a part of nuclear pore complexes (NPC), is essential for kinetochore-microtubule interaction and mitosis. NUP37 has been reported as an oncogene in lung and liver cancers [24–26]. Although NUP37 was demonstrated to be capable of promoting HCC progression via regulation of YAP/TEAD pathway [24], the clinical significance of NUP37 and epigenetic mechanism of NUP37 expression in HCC cases remain largely unknown. Our work comprehensively revealed that NUP37 overexpressed in both clinical HCC samples and human hepatoma cell lines. We also determined that NUP37 overexpression significantly correlated with worse OS in patients with primary HCC from 3 large independent cohorts (Fig. 4D). In addition, multivariate cox survival analysis indicated that NUP37 overexpression was a novel independent risk factor for OS of patients with primary HCC.

Unlike the demethylation profiles of MCM2 in HCC, we found that DNA methylation pattern of NUP37 promoter region in clinical HCC samples was similar with that in adjacent nontumor samples. Interestingly, in comparison with the DNA methylation levels of NUP37 promoter-associated sites, promoter-unassociated sites (cg24826236, cg08316365 and cg08085165) had markedly higher methylation levels (Fig. 8A–C). Furthermore, correlation analysis indicated that DNA methylation level of MCM2 enhancer (cg08889930) significantly negatively correlated with NUP37 mRNA expression in HCC, which indicated that demethylation at cg08889930 indirectly regulating NUP37 expression at transcriptional level. In addition, we found that decitabine can upregulate NUP37 expression by inducing hypomethylation at genome-scale. Inversely, NUP37 expression can be suppressed in HCC cells after treated with KDM5 inhibitor CPI-455. All in all, these finding implied that NUP37 is a potential epigenetic therapy target for HCC patients.

In summary, our work demonstrated that the MCM2 or NUP37 overexpression correlates with worse clinical outcome for patients with primary HCC from 3 large independent cohorts. Demethylation at MCM2 enhancer was a common event in HCC patients. Demethylation at enhancer significantly upregulates MCM2 or NUP37 expression. NUP37 and MCM2 are potential epigenetic therapy targets for HCC patients.

## Supplementary Information

**Additional file 1: Fig. S1.** Representative H&E staining of HCC and paired peri-tumor tissue. **Fig. S2.** Kaplan–Meier survival analysis was performed to investigate the impact of the methylation level of MCM2 enhancer (cg08889930 site) on overall survival of patients from TCGA cohort (n = 374). *p* is based on log-rank test. **Fig. S3.** Validation of the methylation pattern of the indicated CpG islands. (A) Results from pyrosequencing showing that the methylated percentage of CpG sites of MCM2 enhancer region (cg0889930 site) was significantly lower in HCC samples than paired nontumor samples. (B) Pyrosequencing results showing that the methylated percentage of CpG sites of CG0350244 was similar between HCC and than paired nontumor samples. (C) Pyrosequencing results showing that the methylated percentage of CpG sites of CG03502446 was significantly higher in HCC samples than in paired nontumor samples. (D) Box plot showing the mean methylated percentage of CpG sites at NUP37 promoter region was significantly higher in HCC as compared with paired nontumor samples. **Table S1.** Clinicopathological Features of HCC Patients (n = 300). **Table S2.** Primer sequences and location of CpG sites tested using bisulfite pyrosequencing

**Additional file 2. ** The raw reports of pyrosequencing.

## Data Availability

Public omics datasets included in this study are available in The Cancer Genome Atlas (TCGA-LIHC), the National Omics Data Encyclopedia (OEP000321), Gene Expression Omnibus (GSE57957, GSE56588), or ArrayExpress (E-MTAB-4171, E-MTAB-4169) by searching the project name. The raw reports of pyrosequencing in this study are presented in Additional file 2.

## Abbreviations

MCM2: Minichromosome maintenance complex component 2
NUP37: Nucleoporin 37
HCC: Hepatocellular carcinoma
TCGA: The Cancer Genome Atlas
NODE: National Omics Data Encyclopedia
AFP: α-Fetoprotein
DEGs: Differentially expressed genes
DEPs: Differentially expressed proteins
DMRs: Differential methylation regions
TMAs: Tissue microarrays
IHC: Immunohistochemistry
RT-PCR: Real-time PCR
OS: Overall survival
DNMT: DNA methyltransferase
CpG: Cytosine-phosphate-guanine
KDM5: Histone lysine demethylase 5
