# Correction: Demethylation at enhancer upregulates MCM2 and NUP37 expression predicting poor survival in hepatocellular carcinoma patients

**DOI:** 10.1186/s12967-022-03766-0

**Published:** 2022-12-13

**Authors:** Zengwei Tang, Yuan Yang, Wen Chen, Enliang Li, Tingbo Liang

**Affiliations:** 1 Department of Hepatobiliary and Pancreatic Surgery, The First Affiliated Hospital, School of Medicine, Zhejiang University, 79 Qingchun Road, Hangzhou, 310003 Zhejiang China; 2 Zhejiang Provincial Key Laboratory of Pancreatic Disease, The First Affiliated Hospital, School of Medicine, Zhejiang University, Hangzhou, 310003 Zhejiang China; 3 Zhejiang Provincial Innovation Center for the Study of Pancreatic Diseases, Zhejiang Province, HangzhouZhejiang, 310003 China; 4 Department of Hematology, Peking Union Medical College Hospital, Chinese Academy of Medical Science and Peking Union Medical College, Beijing, 100730 China; 5 Cancer Center, Zhejiang University, Hangzhou, 310058 Zhejiang China; 6 Research Center for Healthcare Data Science, Zhejiang Lab, Hangzhou, 310003 Zhejiang China

## Correction: Journal of Translational Medicine (2022) 20:49 https://doi.org/10.1186/s12967-022-03249-2

Following publication of the original article [1], we have been notified that the Figure 2 was published incorrectly. Also, the Figures 2 and 7 captions should be extended and Funding note needs to be corrected.

They should be as follows:

**Fig. 2:**
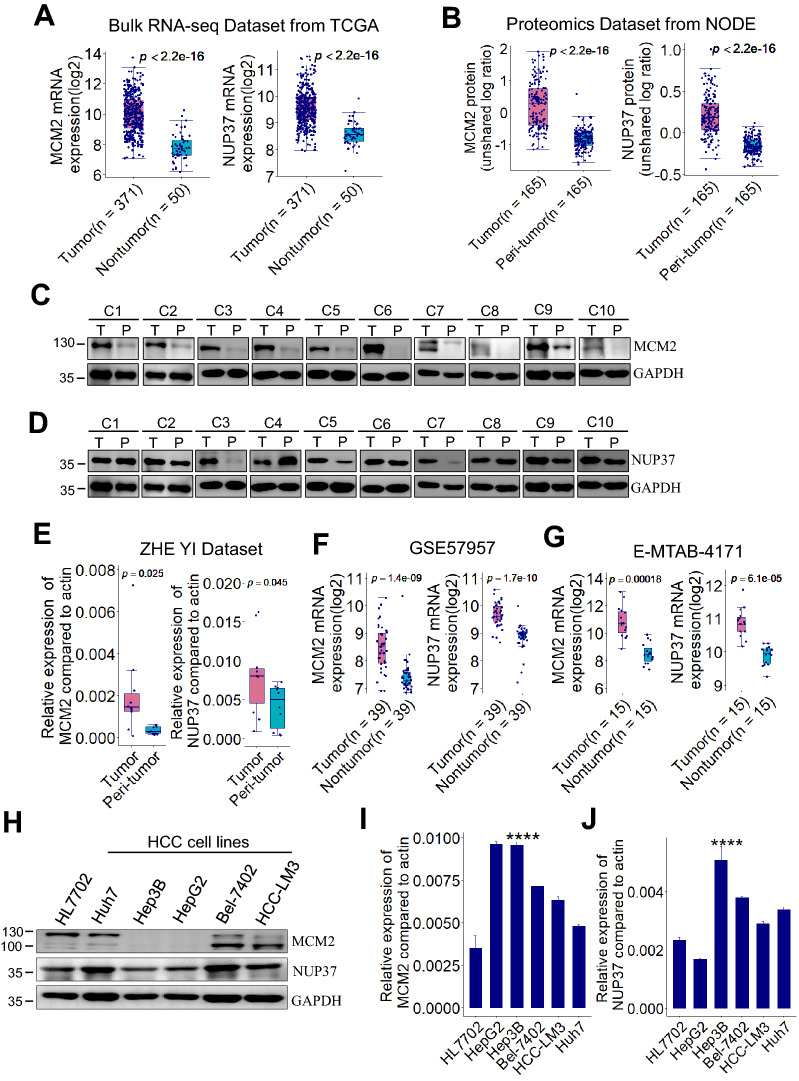
MCM2 and NUP37 are upregulated in human HCC. **A** Box plots showing MCM2 and NUP37 mRNA expression levels between HCC and nontumor samples from TCGA dataset (TCGA-LIHC, n = 421). **B** Box plots showing MCM2 and NUP37 protein expression levels between HCC and peri-tumor samples from NODE dataset (OPE00321, n = 330). *p* value is based on Wilcoxon test. **C**, **D** Representative immunoblots of 10 pairs of HCC sample lysates showing that MCM2 and NUP37 were overexpressed in HCC. The MCM2, NUP37, and GAPDH bands of each paired sample group were acquired in the same gel. **E** MCM2 and NUP37 mRNA expression levels were determined by RT-PCR in 10 pairs of HCC samples. *p < 0.05, **p < 0.01 by paired Student’s t-test. **F**, **G** Box plots showing MCM2 and NUP37 mRNA expression levels between HCC and the paired nontumor tissues samples from GSE57957 and E-MTAB-4171 dataset respectively. **H**–**J** MCM2 and NUP37 expression were detected by immunoblot and RT-PCR in human HCC cells and normal liver cells. HCC, hepatocellular carcinoma; TCGA, ****p < 0.0001, based on ANOVA. TCGA, The Cancer Genome Atlas; LIHC, liver hepatocellular carcinoma; NODE, National Omics Data Encyclopedia; RT-PCR, real-time PCR

**Fig. 7** Demethylation at enhancer region (cg08889930) significantly negatively correlated with NUP37 mRNA expression. **A** Diagram showing the CpG islands in the promoter region of human NUP37 gene. **B** Heatmap showing overall methylation patterns in the DNA sequence of human NUP37 gene. **C** Correlation heatmap visualizing the relationship between NUP37 mRNA expression and the methylation level of CpG island at DNA sequence of NUP37 gene. **D–F** Scatter plot showing that NUP37 mRNA expression significantly negatively correlated with the methylation level of cg08889930 in HCC patients from 3 independent datasets. **G** NUP37 mRNA expression in Huh7 cells significantly increased after decitabine treatment ***p < 0.001, based on ANOVA. **H** NUP37 mRNA expression in Huh7 cells significantly reduced after treatment with CPI-455. ****p < 0.0001, based on ANOVA. **I–J** Representative immunoblot of HCC cells (Huh7) lysates showing that decitabine increased NUP37 protein expression and CPI-455 decreased NUP37 protein expression. The NUP37, GAPDH and MCM2 (Fig 6I-J) bands of Huh7 cell lysates were obtained from same gel.

**Funding note:** This study was funded by the grants from National Key Research and Development Program (No 2019YFC1316000) and the Zhejiang Provincial Program for the Cultivation of High-level Innovative Health Talents. The funders played no roles in the study design, the collection, analysis and interpretation of data, the writing of the report, and the decision to submit the article for publication.
