# Supplementary material for: Demethylation at enhancer upregulates MCM2 and NUP37 expression predicting poor survival in hepatocellular carcinoma patients
Source: J Transl Med. 2022 Jan 29;20:49. doi: 10.1186/s12967-022-03249-2 (PMC8800332; doi:10.1186/s12967-022-03249-2)
Supplement: Supplementary file 1 — Additional file 1: Fig. S1. Representative H&E staining of HCC and paired peri-tumor tissue. Fig. S2. Kaplan–Meier survival analysis was performed to investigate the impact of the methylation level of MCM2 enhancer (cg08889930 site) on overall survival of patients from TCGA cohort (n = 374). p is based on log-rank test. Fig. S3. Validation of the methylation pattern of the indicated CpG islands. (A) Results from pyrosequencing showing that the methylated percentage of CpG sites of MCM2 enhancer region (cg0889930 site) was significantly lower in HCC samples than paired nontumor samples. (B) Pyrosequencing results showing that the methylated percentage of CpG sites of CG0350244 was similar between HCC and than paired nontumor samples. (C) Pyrosequencing results showing that the methylated percentage of CpG sites of CG03502446 was significantly higher in HCC samples than in paired nontumor samples. (D) Box plot showing the mean methylated percentage of CpG sites at NUP37 promoter region was significantly higher in HCC as compared with paired nontumor samples. Table S1. Clinicopathological Features of HCC Patients (n = 300). Table S2. Primer sequences and location of CpG sites tested using bisulfite pyrosequencing [file 12967_2022_3249_MOESM1_ESM.pdf]

Supplementary figure legends

Fig. S1. Representative H&E staining of HCC and paired peri-tumor tissue.

Fig. S2. Kaplan-Meier survival analysis was performed to investigate the impact of the methylation level of MCM2 enhancer (cg08889930 site) on overall survival of patients from TCGA cohort (n = 374). ***p*** **is based on log-rank test.**

Fig. S3. Validation of the methylation pattern of the indicated CpG islands. (A) Results from pyrosequencing showing that the methylated percentage of CpG sites of MCM2 enhancer region (cg0889930 site) was significantly lower in HCC samples than paired nontumor samples. (B) Pyrosequencing results showing that the methylated percentage of CpG sites of CG0350244 was similar between HCC and than paired nontumor samples. (C) Pyrosequencing results showing that the methylated percentage of CpG sites of CG03502446 was significantly higher in HCC samples than in paired nontumor samples. (D) Box plot showing the mean methylated percentage of CpG sites at NUP37 promoter region was significantly higher in HCC as compared with paired nontumor samples.

Fig. S1

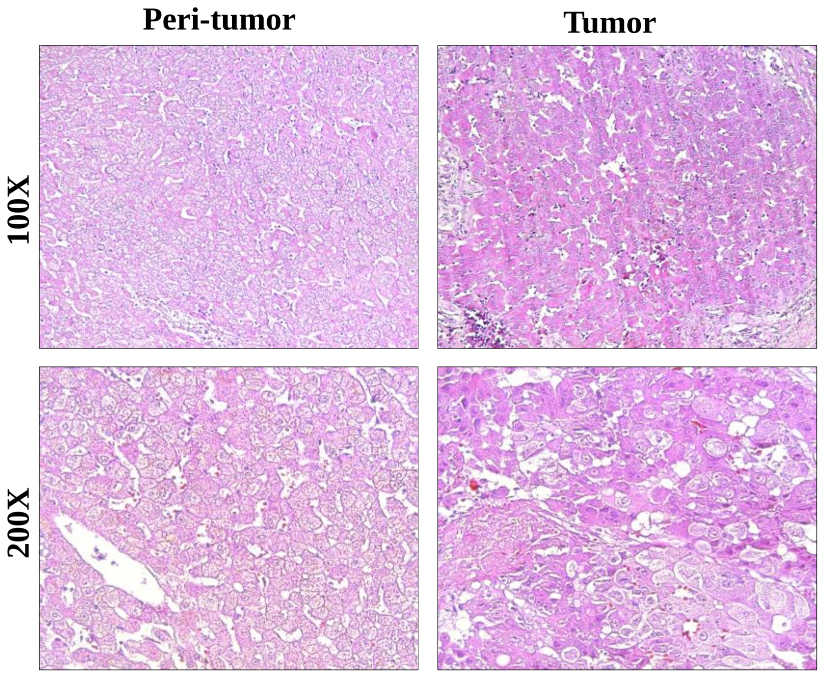

Fig. S2

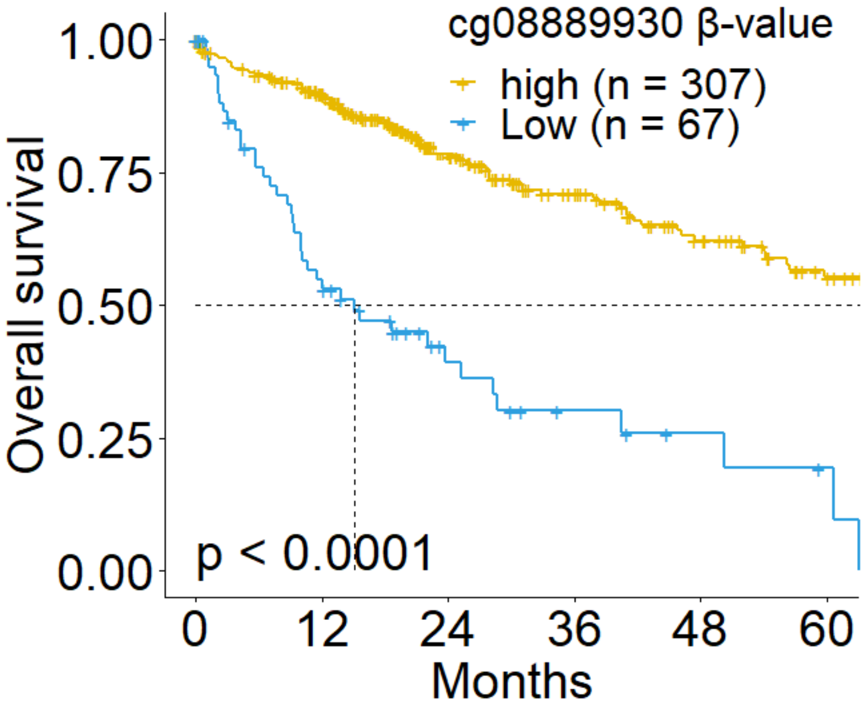
cg08889930 β-value
high (n = 307)
Low (n = 67)
Overall survival
1.00
0.75
0.50
0.25
0.00
p < 0.0001
0
12
24
36
48
60
Months

Fig. S3

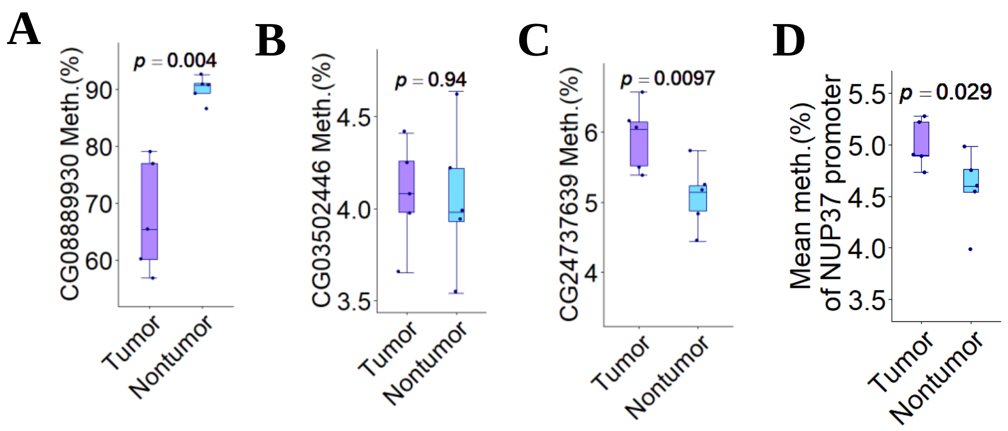
A
B
C
D
p = 0.004
p = 0.94
p = 0.0097
p = 0.029
CG08889930 Meth.(%)
CG03502446 Meth.(%)
CG24737639 Meth.(%)
Mean meth.(%) of NUP37 promoter
Tumor
Nontumor

Supplementary Table 1. Clinicopathological Features of HCC Patients (n = 300)	
**Age, median (range), year**	61 (18-88)
**Gender (F: M), n (%)**	39 (13.0): 261 (87.0)
**Histological grade, n (%)**	
**Low/Low-moderate**	67 (22.3)
**Moderate/Moderate-well**	207 (69.0)
**Well**	26 (8.7)
**Tumor size, mean (range), cm**	4.8 (1.0-18.0)
**AFP, mean (range), ug/L**	2434 (1-200000)
**ALT, mean (range), U/L**	46 (6-702)
**AST, mean (range), U/L**	56 (11-1597)
**T-stage, n (%)**	
**T1a**	63 (21.0)
**T1b**	105 (35.0)
**T2**	90 (30.0)
**T3**	11 (3.7)
**T4**	31(10.3)
**N-stage, n (%)**	
**N0**	291 (97.0)
**N1**	2 (2.0)
**Nx**	1(1.0)
**M-stage, n (%)**	
**M0**	289 (96.3)
**M1**	11 (3.7)
**TNM-stage, n (%)**	
**IA/IB**	63 (21.0)/102 (34.0)
**II**	86 (28.7)
**IIIA/IIIB**	12 (4.0)/24 (8.0)
**IVA/IVB**	5 (1.7)/8 (2.7)
**Hypertension (yes: no), n (%)**	76 (25.3): 224 (74.7)
**Diabetes (yes: no), n (%)**	28 (9.3): 272 (90.7)
**Liver cirrhosis (yes: no), n (%)**	31 (10.3): 269 (89.7)
**Fatty liver (yes: no), n (%)**	32 (10.7): 268 (89.3)
**Hepatitis B virus (yes: no), n (%)**	221 (73.7): 79 (26.3)

HCC, hepatocellular carcinoma; F, female; M, male; AFP, α-fetoprotein; ALT, alanine aminotransferase; AST, aspartate aminotransferase;

Supplementary Table 2. Primer sequences and location of CpG sites tested using bisulfite pyrosequencing

Sequence name	primer	Sequence (5' → 3')	Sequence to Analyze	Strand	CpG site location (hg19)
**C**G08889930	Forward	/5'Biotin/TTTGTTTTGGTGGTTTTTTTATGTTAT	RCAAAATATT ATAATAAAAA TAACAT	+	chr3:127317798 (CG1)
	Reverse	CTCCCCTTAAACATTAAACCTATTTCA			
	Sequencing	ATTTCAACACACACAATAC			
CG03502446 & CG24737639	Forward	GGAAGGAAGAATTTTTGGGAATAG	GGTTGGGYGG TYGGTAGAAA AGTATTT	+	chr12:102513777 (CG2)
	Reverse	/5'Biotin/CCAAAAAAAACCAACCTACCTCTAA			chr12:102513781 (CG3)
	Sequencing	TGGGAATAGAGTGGTAA			

/5Biosg/ = 5’ biotin added
