# Supplementary material for: Demethylation at enhancer upregulates MCM2 and NUP37 expression predicting poor survival in hepatocellular carcinoma patients
Source: J Transl Med. 2022 Jan 29;20:49. doi: 10.1186/s12967-022-03249-2 (PMC8800332; doi:10.1186/s12967-022-03249-2)

CpG Pyrogram Report Run: 20210505 B

Well: B3
Assay: 1.CG08889930-S
Sample ID: P1
Sequence to analyze: RCAAAATATTATAATAAAAATAACAT

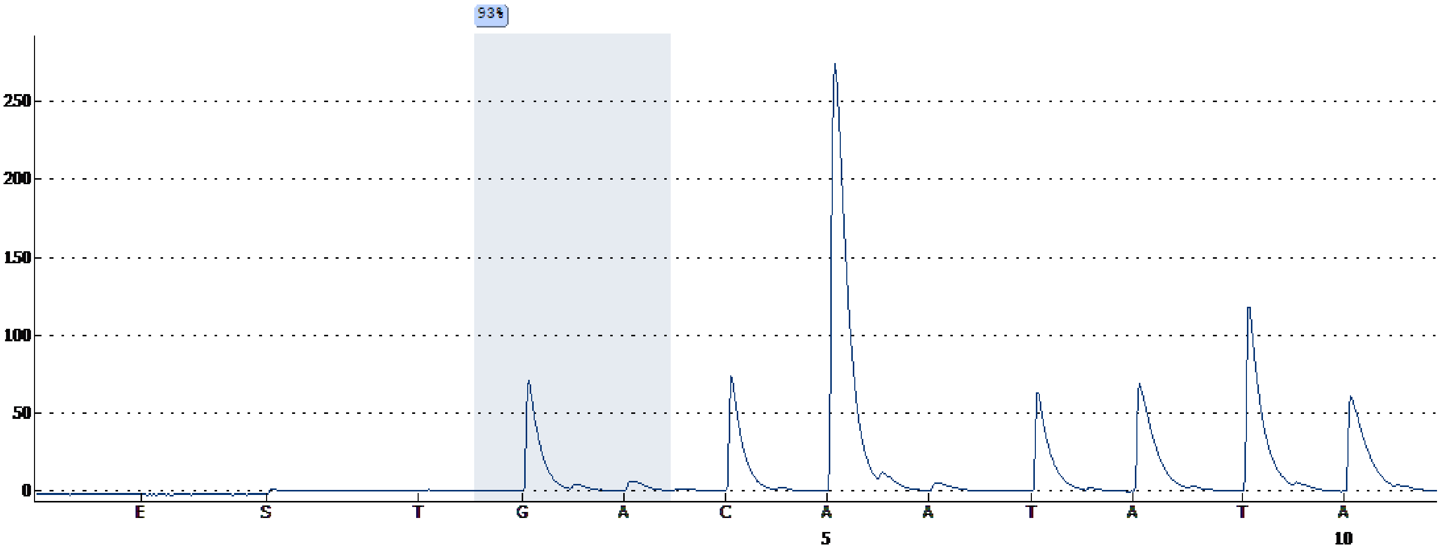

Well: B4
Assay: 1.CG08889930-S
Sample ID: P3
Sequence to analyze: RCAAAATATTATAATAAAAATAACAT

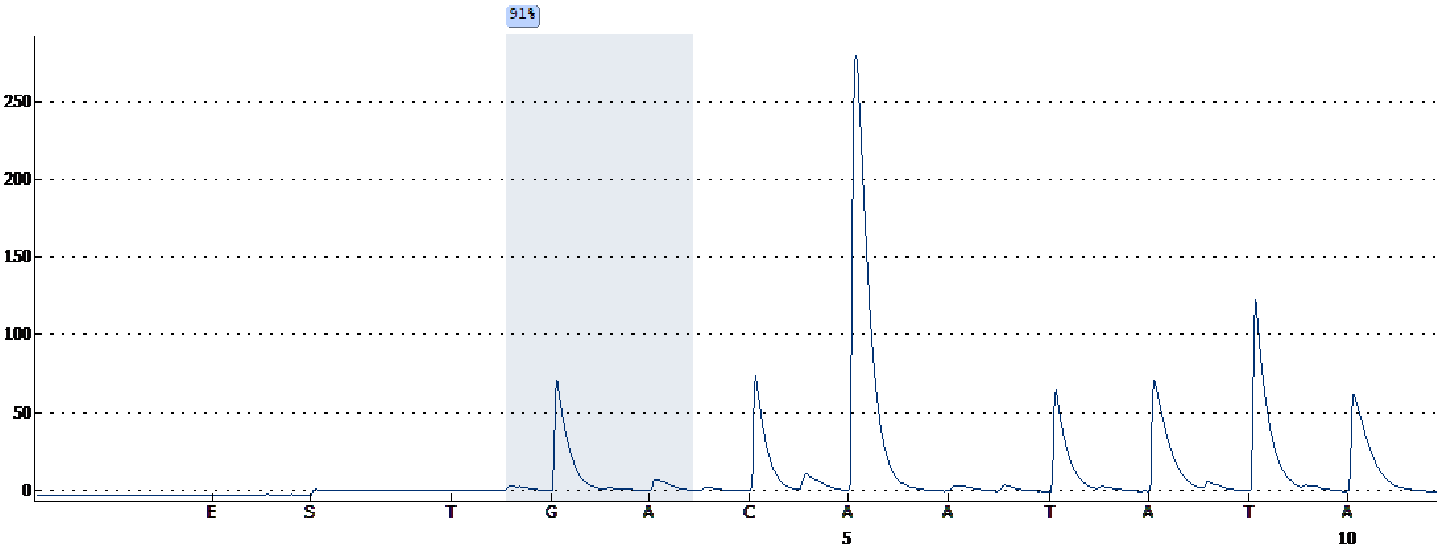

Well: B5
Assay: 1.CG08889930-S
Sample ID: P4
Sequence to analyze: RCAAAATATTATAATAAAAATAACAT

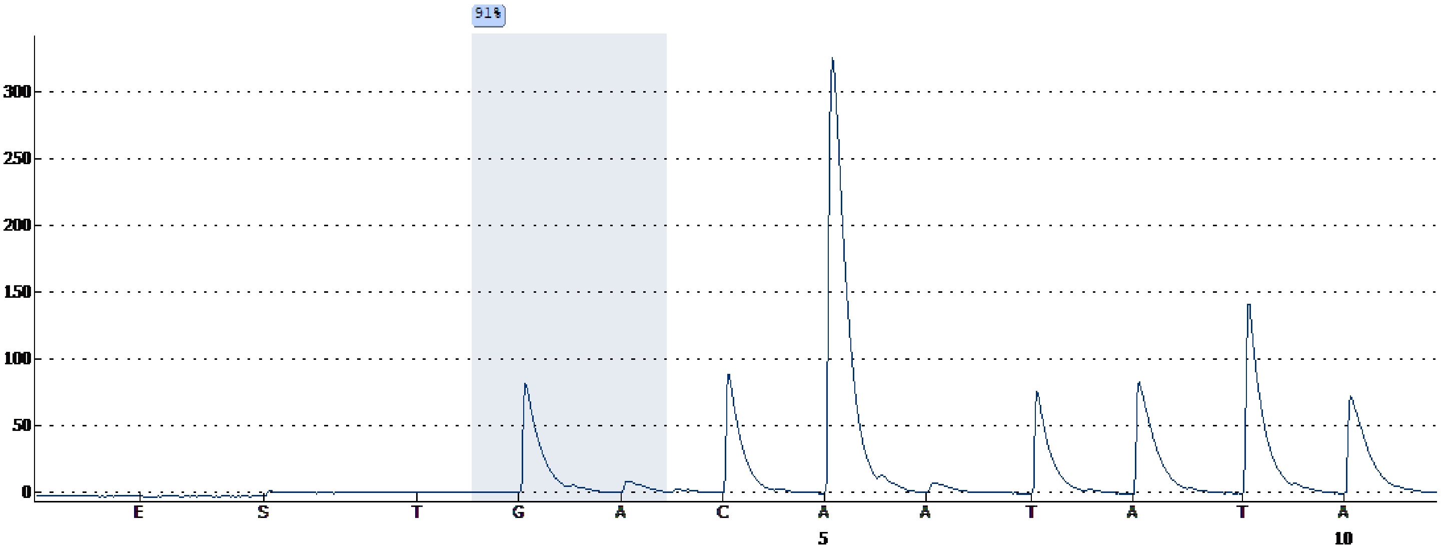

CpG Pyrogram Report Run: 20210505 B

Well: B6
Assay: 1.CG08889930-S
Sample ID: P5
Sequence to analyze: RCAAAATATTATAATAAAAATAACAT

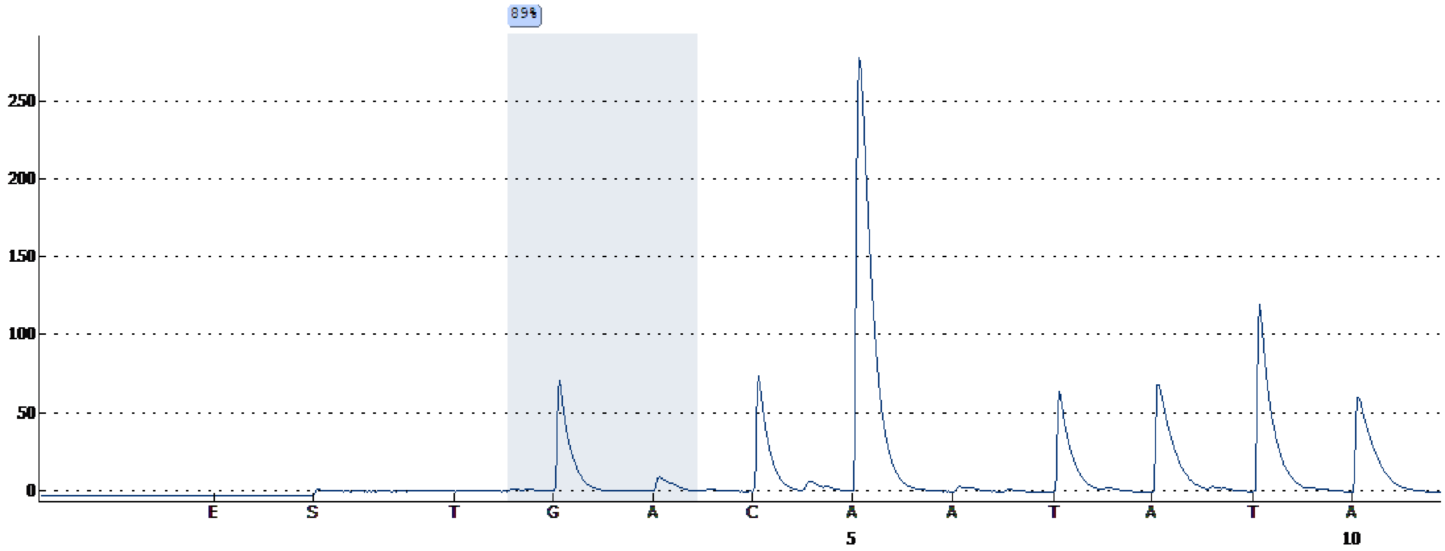

Well: B7
Assay: 1.CG08889930-S
Sample ID: P6
Sequence to analyze: RCAAAATATTATAATAAAAATAACAT

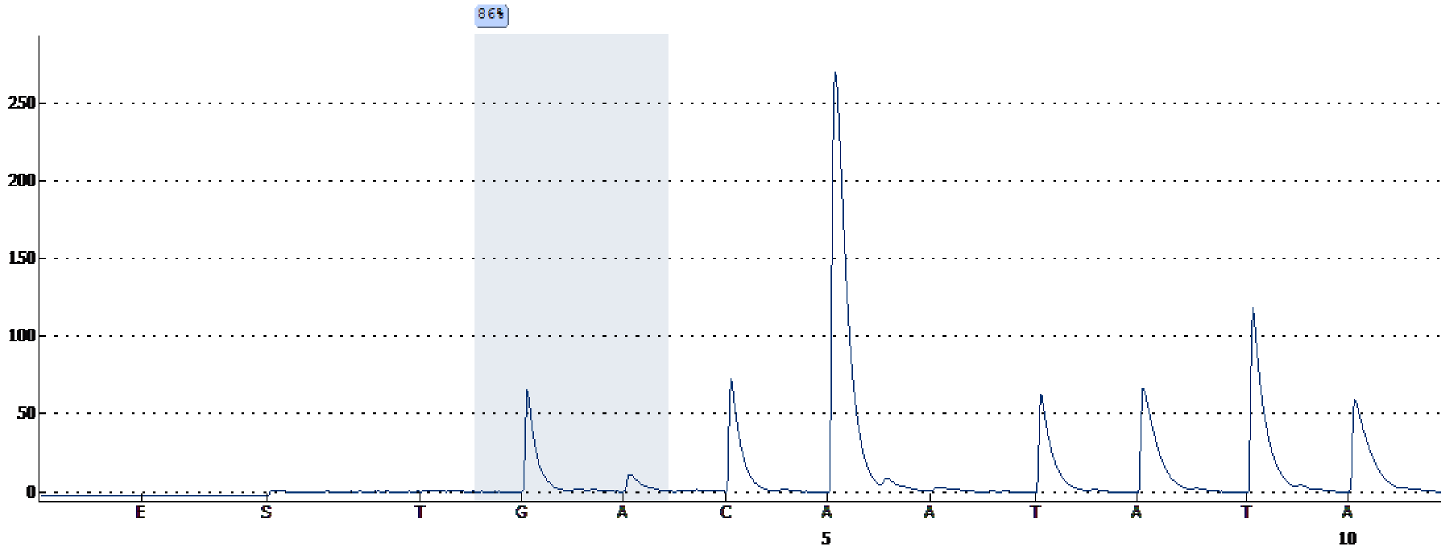

Well: B8
Assay: 1.CG08889930-S
Sample ID: T1
Sequence to analyze: RCAAAATATTATAATAAAAATAACAT

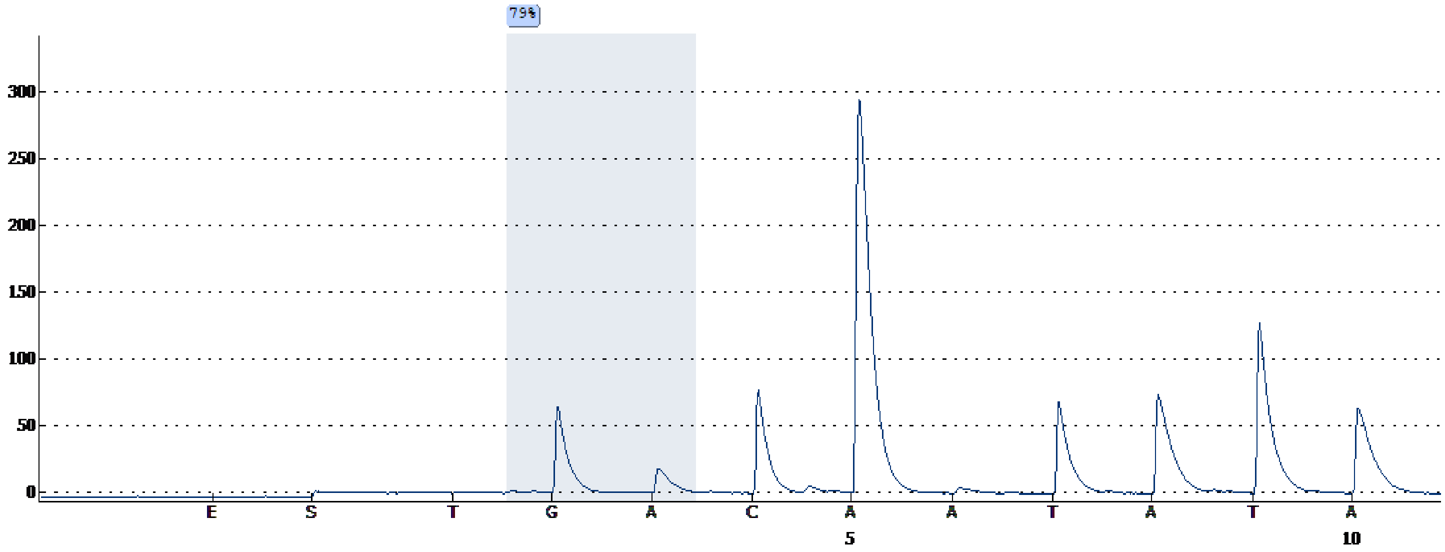

CpG Pyrogram Report Run: 20210505 B

Well: B9
Assay: 1.CG08889930-S
Sample ID: T3
Sequence to analyze: RCAAAATATTATAATAAAAATAACAT

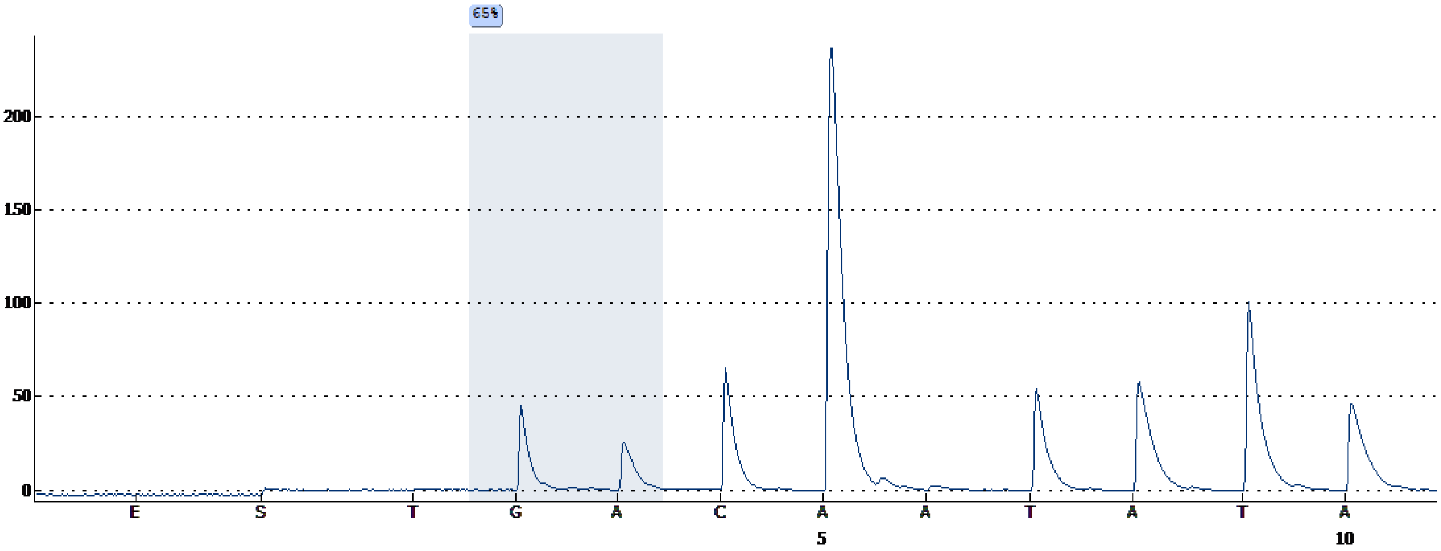

Well: B10
Assay: 1.CG08889930-S
Sample ID: T4
Sequence to analyze: RCAAAATATTATAATAAAAATAACAT

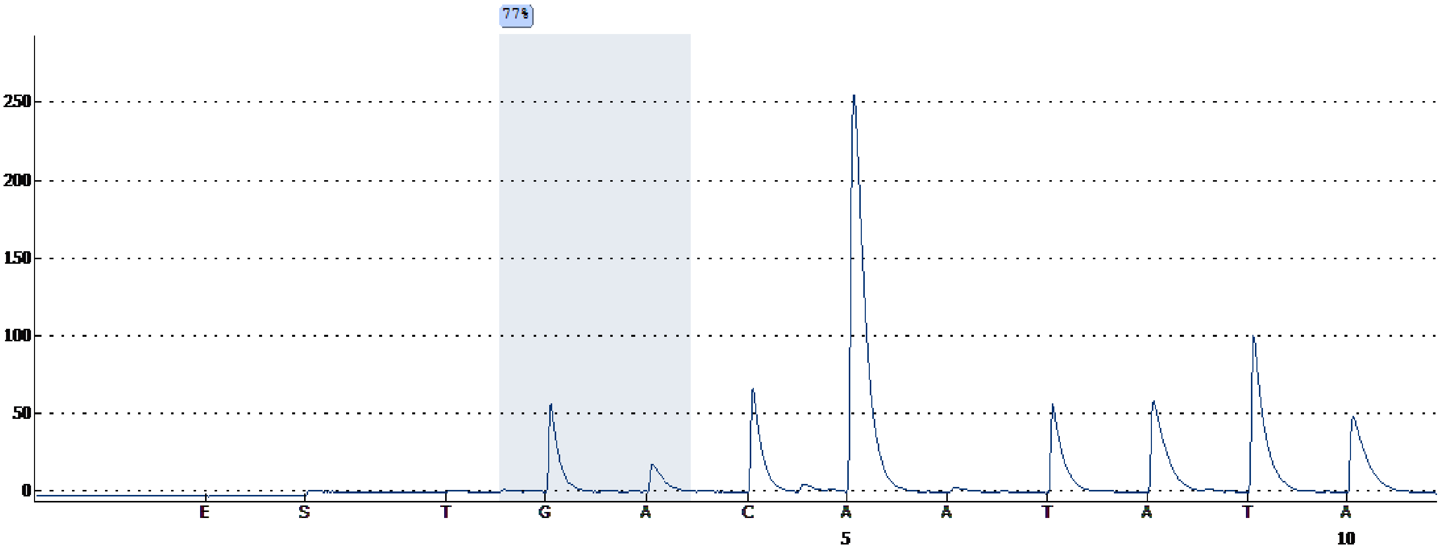

Well: B11
Assay: 1.CG08889930-S
Sample ID: T5
Sequence to analyze: RCAAAATATTATAATAAAAATAACAT

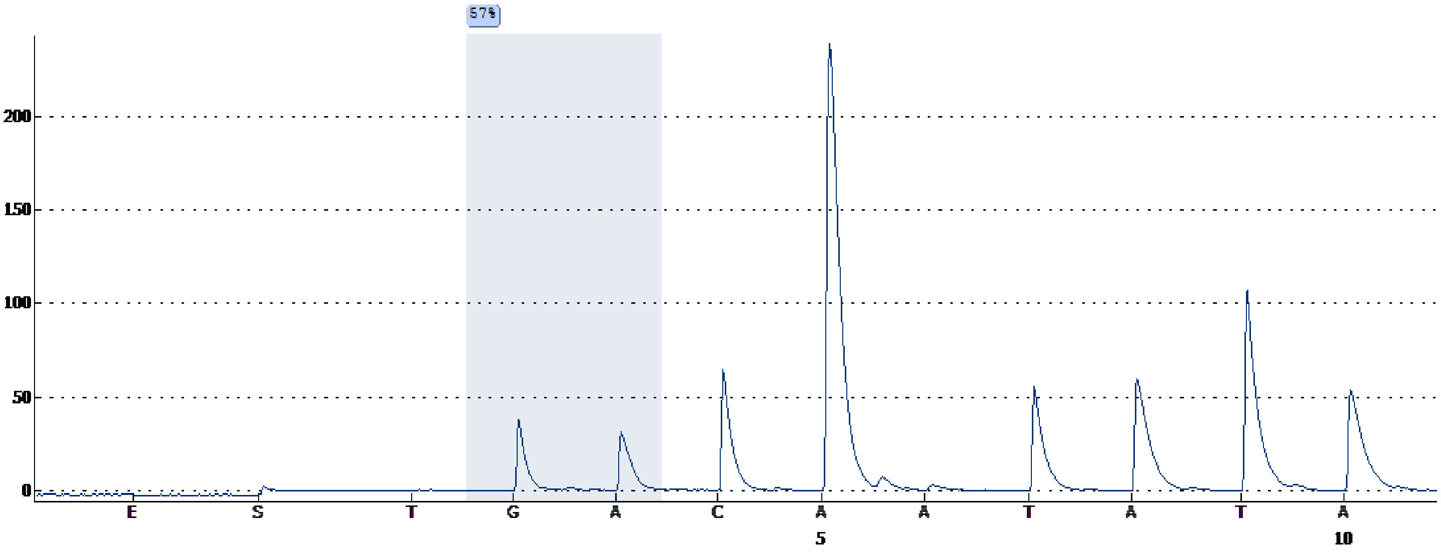

CpG Pyrogram Report Run: 20210505 B

Well: B12
Assay: 1.CG08889930-S
Sample ID: T6
Sequence to analyze: RCAAAATATTATAATAAAAATAACAT

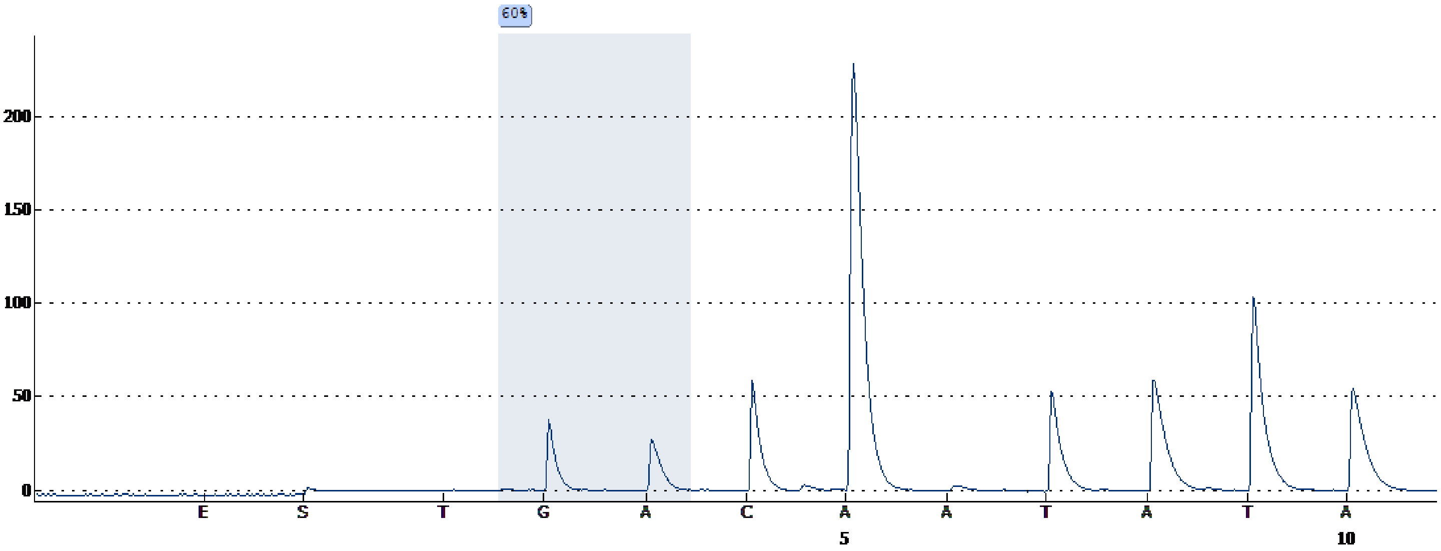

CpG Pyrogram Report Run: 20210505 B

Well: C1
Assay: 2.CG03502446-S
Sample ID: P1
Sequence to analyze: GGTTGGGYGGTYGGTAGAAAAGTATTT

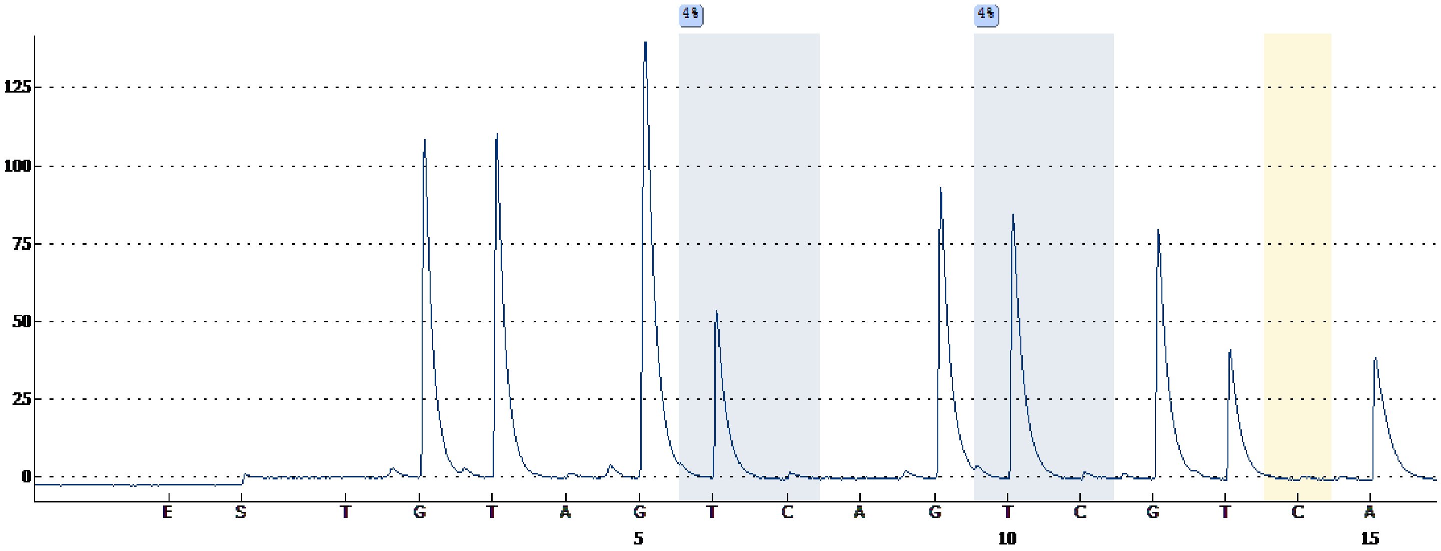

Well: C2
Assay: 2.CG03502446-S
Sample ID: P3
Sequence to analyze: GGTTGGGYGGTYGGTAGAAAAGTATTT

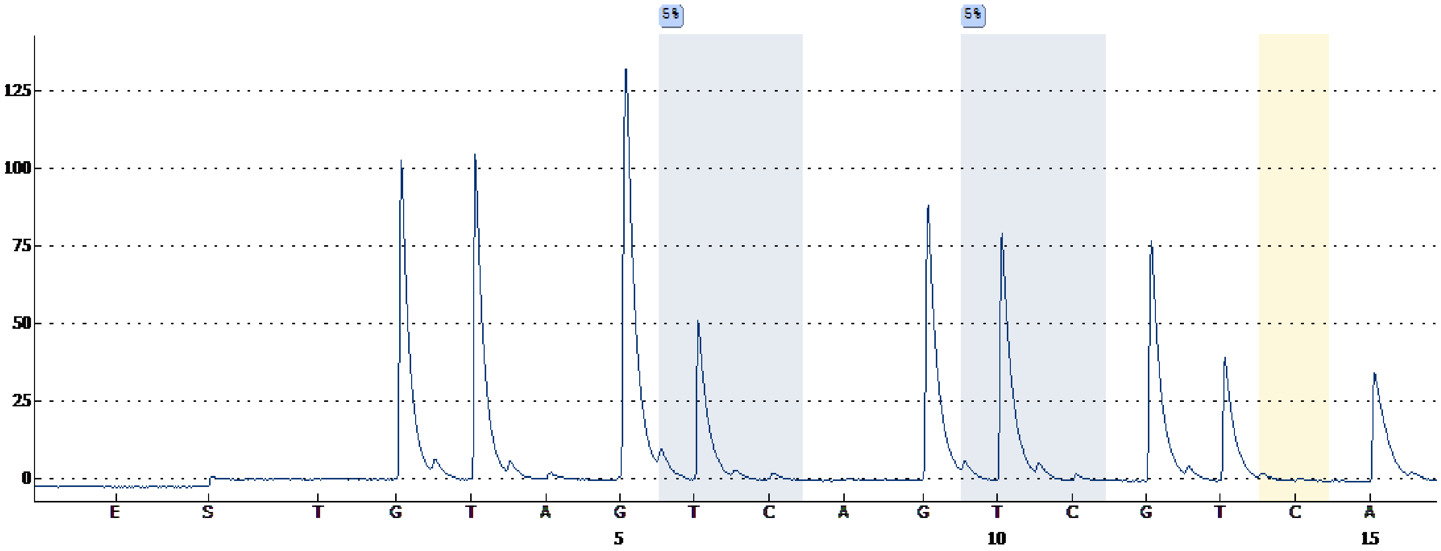

Well: C3
Assay: 2.CG03502446-S
Sample ID: P4
Sequence to analyze: GGTTGGGYGGTYGGTAGAAAAGTATTT

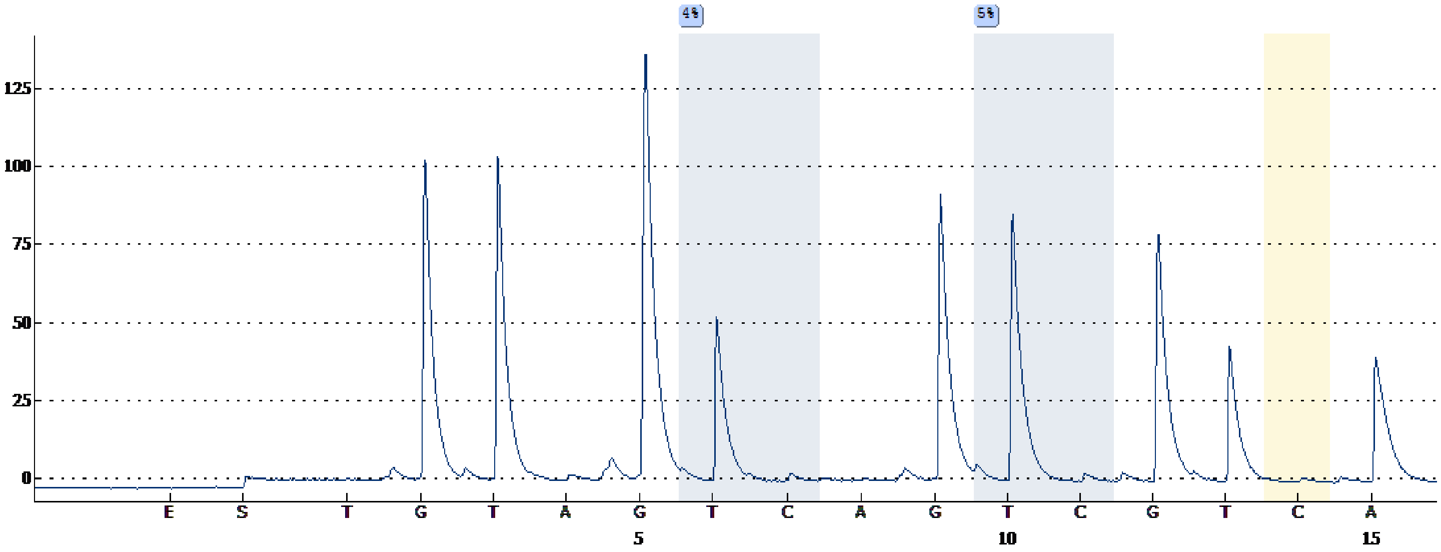

CpG Pyrogram Report Run: 20210505 B

Well: C4
Assay: 2.CG03502446-S
Sample ID: P5
Sequence to analyze: GGTTGGGYGGTYGGTAGAAAAGTATTT

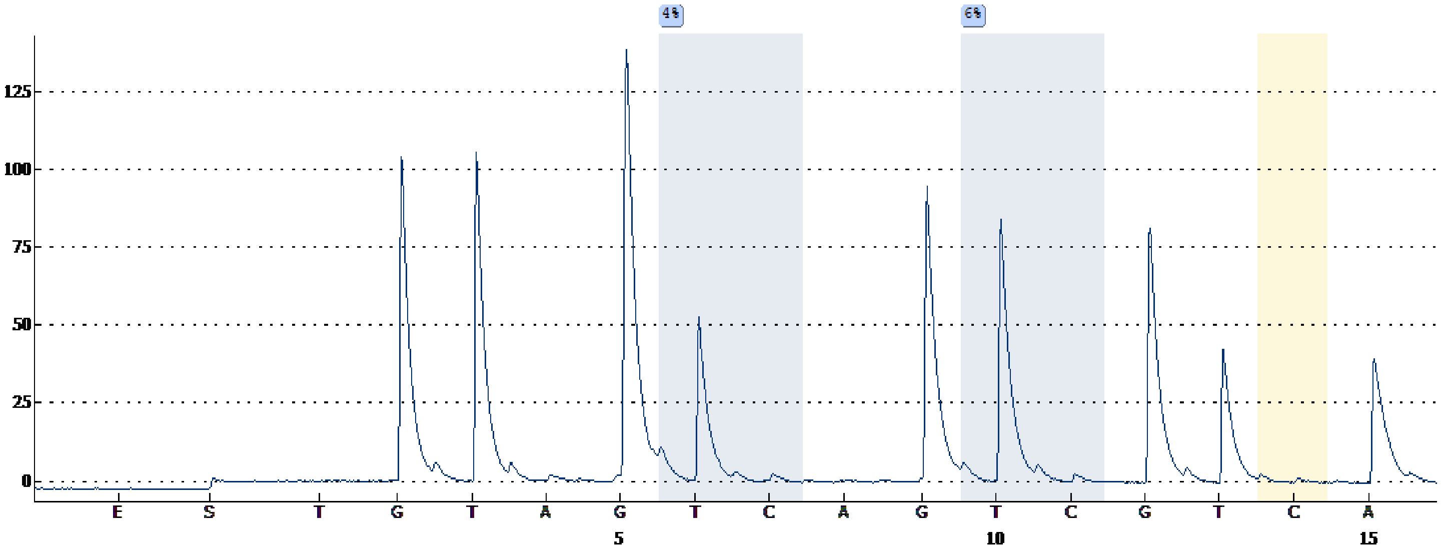

Well: C5
Assay: 2.CG03502446-S
Sample ID: P6
Sequence to analyze: GGTTGGGYGGTYGGTAGAAAAGTATTT

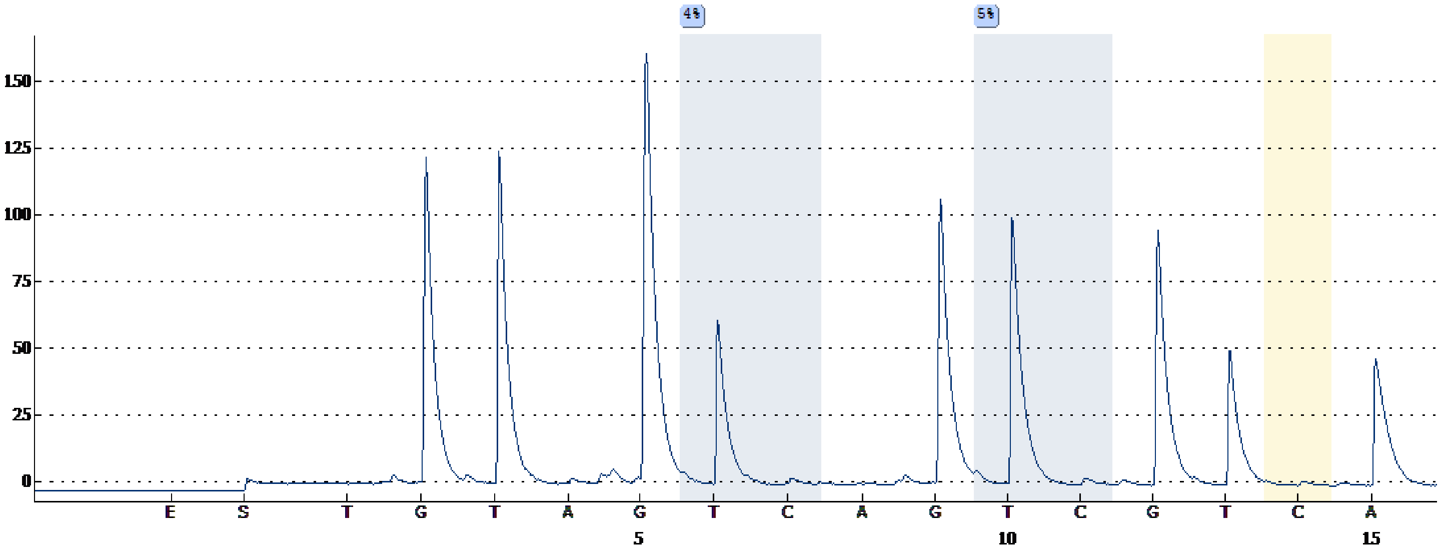

Well: C6
Assay: 2.CG03502446-S
Sample ID: T1
Sequence to analyze: GGTTGGGYGGTYGGTAGAAAAGTATTT

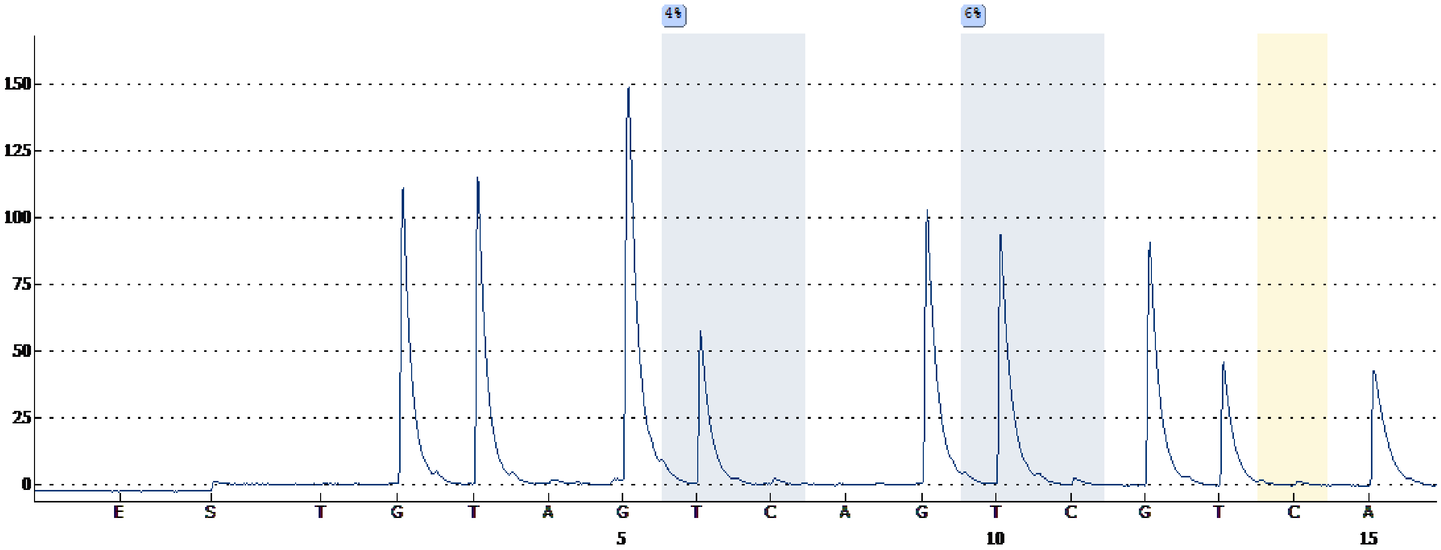

CpG Pyrogram Report Run: 20210505 B

Well: C7
Assay: 2.CG03502446-S
Sample ID: T3
Sequence to analyze: GGTTGGGYGGTYGGTAGAAAAGTATTT

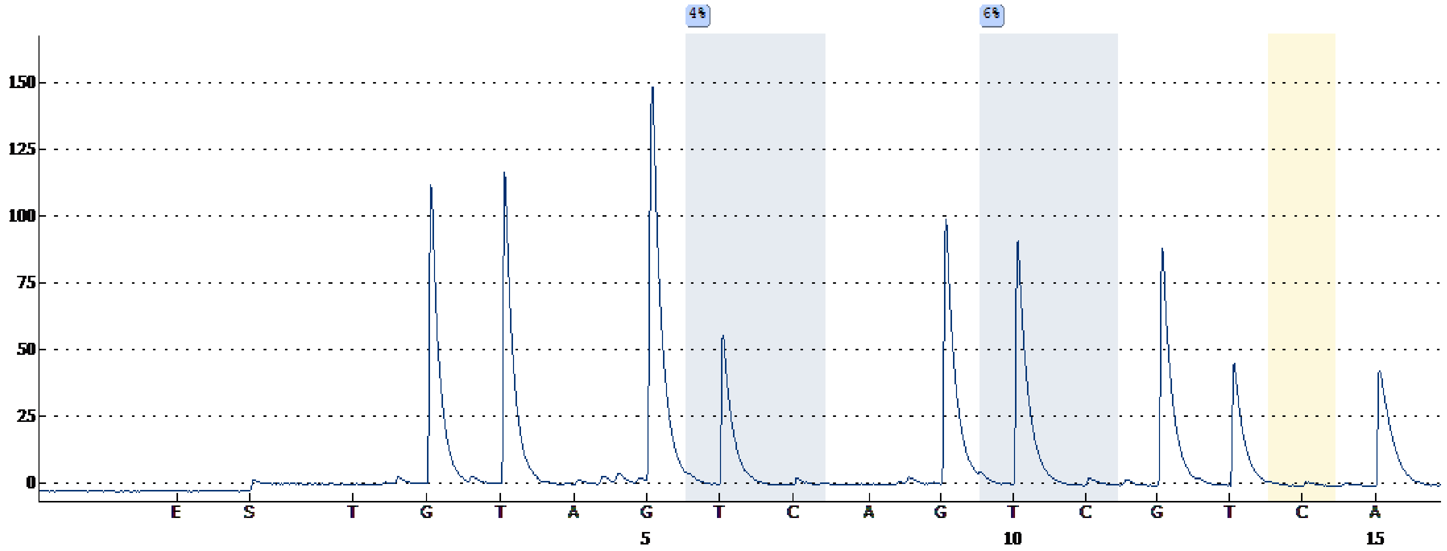

Well: C8
Assay: 2.CG03502446-S
Sample ID: T4
Sequence to analyze: GGTTGGGYGGTYGGTAGAAAAGTATTT

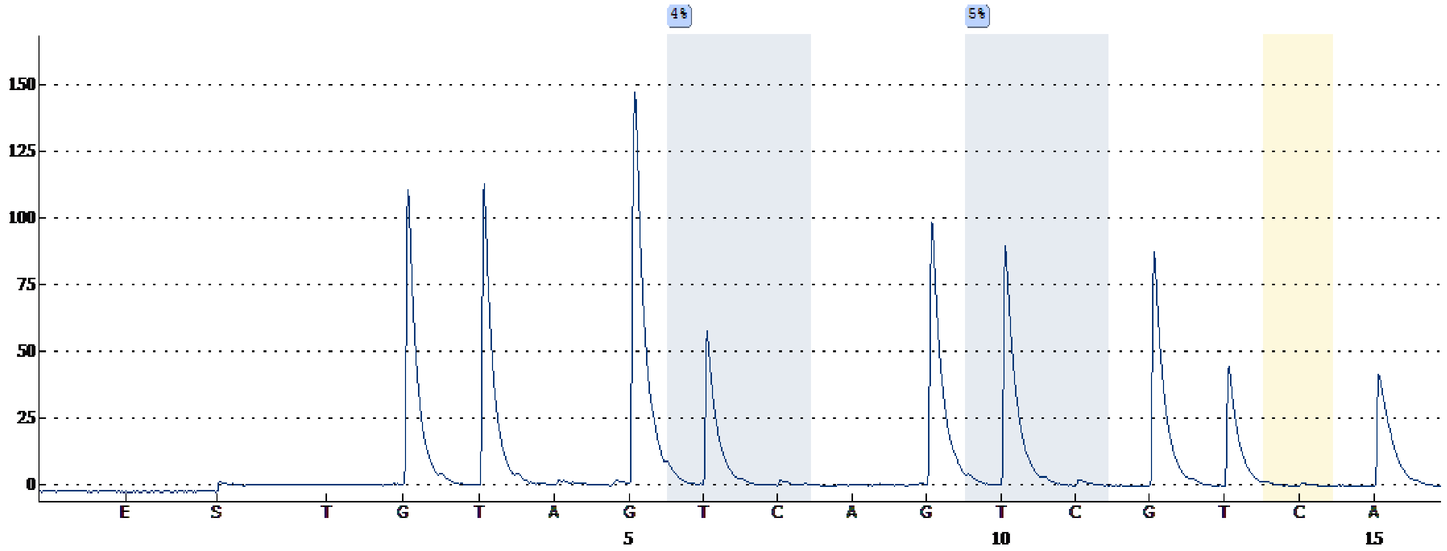

Well: C9
Assay: 2.CG03502446-S
Sample ID: T5
Sequence to analyze: GGTTGGGYGGTYGGTAGAAAAGTATTT

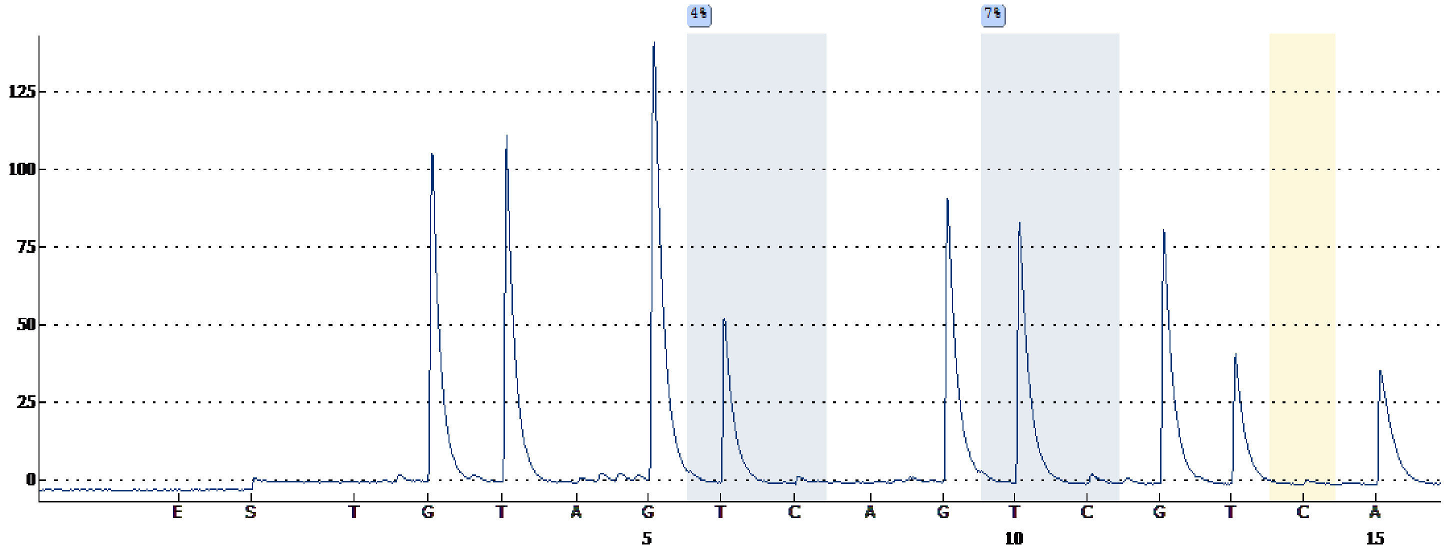

CpG Pyrogram Report Run: 20210505 B

Well: C10
Assay: 2.CG03502446-S
Sample ID: T6
Sequence to analyze: GGTTGGGYGGTYGGTAGAAAAGTATTT

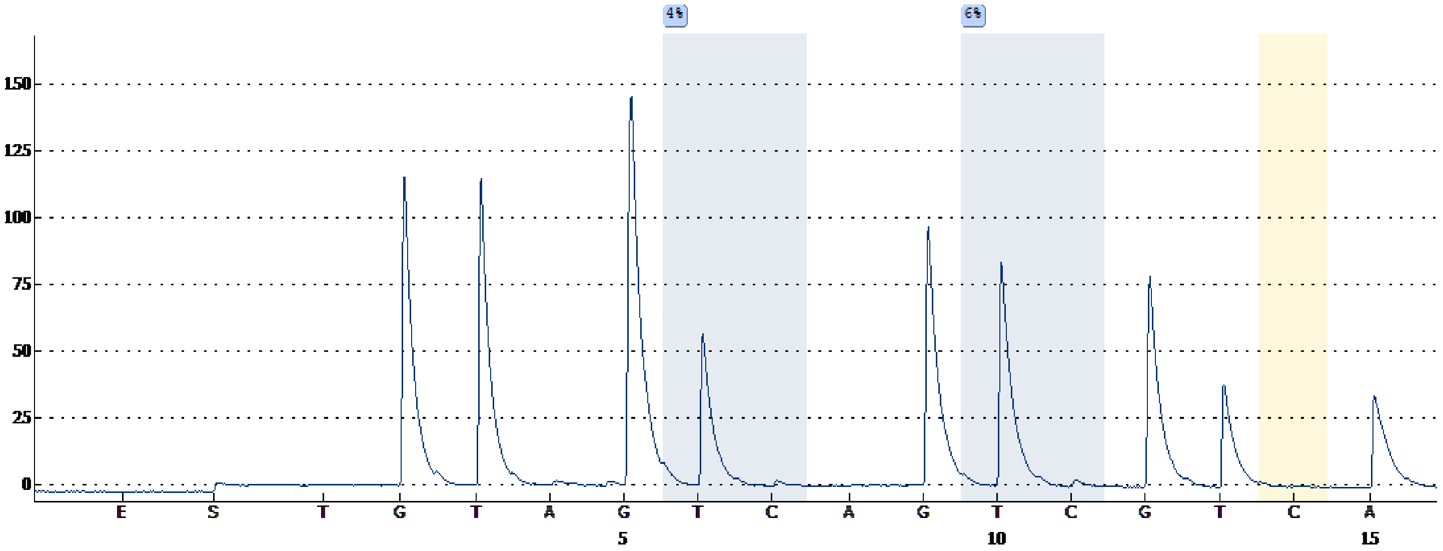

Supplement: Supplementary file 2 — Additional file 2. The raw reports of pyrosequencing. [file 12967_2022_3249_MOESM2_ESM.pdf]
